# Nodal band-off-diagonal superconductivity in twisted graphene superlattices

**DOI:** 10.1038/s41467-023-42471-4

**Published:** 2023-11-06

**Authors:** Maine Christos, Subir Sachdev, Mathias S. Scheurer

**Affiliations:** 1 Department of Physics, Harvard University, Cambridge, MA 02138 USA; 2 Institute for Theoretical Physics, University of Innsbruck, Innsbruck, A-6020 Austria; 3 Institute for Theoretical Physics III, University of Stuttgart, 70550 Stuttgart, Germany

**Keywords:** Superconducting properties and materials, Electronic properties and materials

## Abstract

The superconducting state and mechanism are among the least understood phenomena in twisted graphene systems. Recent tunneling experiments indicate a transition between nodal and gapped pairing with electron filling, which is not naturally understood within current theory. We demonstrate that the coexistence of superconductivity and flavor polarization leads to pairing channels that are guaranteed by symmetry to be entirely band-off-diagonal, with a variety of consequences: most notably, the pairing invariant under all symmetries can have Bogoliubov Fermi surfaces in the superconducting state with protected nodal lines, or may be fully gapped, depending on parameters, and the band-off-diagonal chiral *p*-wave state exhibits transitions between gapped and nodal regions upon varying the doping. We demonstrate that band-off-diagonal pairing can be the leading state when only phonons are considered, and is also uniquely favored by fluctuations of a time-reversal-symmetric intervalley coherent order motivated by recent experiments. Consequently, band-off-diagonal superconductivity allows for the reconciliation of several key experimental observations in graphene moiré systems.

## Abstract (web summary)

The authors theoretically study superconductivity in twisted-bilayer and twisted-trilayer graphene, finding that flavor polarization allows for Cooper pairing in which the pairs consist of electrons in different bands. Both intervalley phonons and fluctuations of a time-reversal-symmetric intervalley coherent order can favor such pairing.

## Introduction

The fascinating physics^1,2^ of correlated graphene moiré superlattices, such as twisted-bilayer (TBG) and twisted-trilayer graphene (TTG), has generated extensive efforts to uncover the mysteries of their phase diagrams. Much progress has been made towards understanding their normal-state physics, including the correlated insulating phases^3–18^ and the reset behavior^19,20^; the latter, which is believed to be associated with the onset of flavor polarization, appears in the same density range and can coexist with superconductivity^13,19–34^. However, the form and symmetry of the superconducting order parameter and the pairing glue are still unknown, despite significant theoretical efforts^27–30,33,35–47^.

Tunneling conductance measurements taken within the superconducting state reveal a V-shaped density of states (DOS)^48,49^ which can become *U*-shaped at other electron concentrations^49^. Setting aside the possibility of thermal fluctuations as origin^50^, this is most naturally interpreted as a transition from nodal to fully gapped superconductivity. For a consistent microscopic theoretical understanding, this provides the following challenges: (i) electron–phonon coupling—a widely discussed^33,35–40^ pairing mechanism in TBG and TTG—will typically mediate an entirely attractive interaction in the Cooper channel, with the leading pairing state that transforms trivially under all symmetries and is thus fully gapped^51,52^. (ii) Even when the low-energy interactions favor an irreducible presentation (IR), e.g., *E* of *C*_3_, with nodal basis functions (*p*- or *d*-wave), the generically fully gapped chiral configuration wins over the nodal nematic one within mean-field. (iii) Even if we assume that the nodal state is energetically favored, e.g., due to significant corrections beyond mean-field^27,53–55^, one is still left to explain why there is a transition to another, fully gapped superconductor upon changing the filling.

**Fig. 1:**
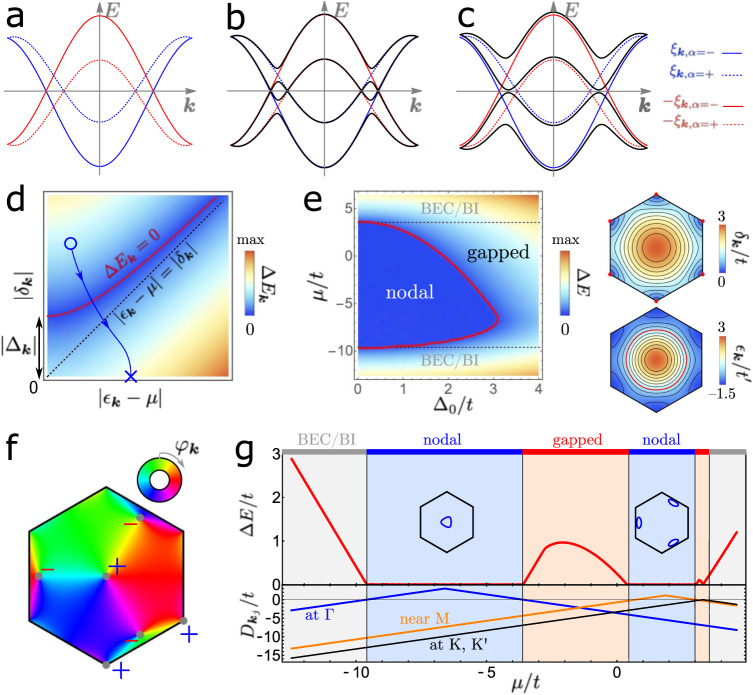
Spectral properties of interband pairing. While for band-diagonal pairing a small superconducting order parameter can immediately open up a gap as time-reversal symmetry guarantees that the associated avoided crossings [gray regions in **a**] in the Bogoliubov spectrum are at the Fermi level, this is not the case for band-off-diagonal pairing (**b**). Here, a sufficiently strong order-parameter value is required to establish a full gap, see **c**. Its ***k*** dependence according to Eq. (3) is shown in **d**, where the red line indicates nodal points. If the band structure has Dirac points, there will be a point on the horizontal axis (blue cross). Consequently, if there is another momentum point located above the red line (blue circle), continuity of the Hamiltonian implies a nodal point on any path connecting the two momenta. **e** Gap of the isotropic *A*_2_ state and *δ*_***k***_, *ϵ*_***k***_ (zeros indicated in red) for the normal-state toy model defined in the text. BEC/BI refers to the Bose-Einstein condensate/band insulator limit. **f** Complex phase ${\varphi }_{{{{{{{{\boldsymbol{k}}}}}}}}}=\arg ({X}_{{{{{{{{\boldsymbol{k}}}}}}}}}+i{Y}_{{{{{{{{\boldsymbol{k}}}}}}}}})$ for leading basis function with small subleading corrections. **g** Shows the gap of the chiral *p*-wave *E*_2_(1, *i*) state with Δ_0_ = 1.5*t* and the value of ${D}_{{{{{{{{{\boldsymbol{k}}}}}}}}}_{j}}: \!\!=| {\delta }_{{{{{{{{{\boldsymbol{k}}}}}}}}}_{j}}| -| {\epsilon }_{{{{{{{{{\boldsymbol{k}}}}}}}}}_{j}}-\mu |$ for ***k***_*j*_ at the three symmetry-in-equivalent vortices in **f** as a function of *μ*. We took ${t}^{{\prime} }=-2.2t,t \, > \, 0$, in **b**, **d**.

In this work, we show that the combination of flavor polarization and the representations of the symmetries in the flat bands of TBG and TTG allow for pairing channels that are completely off-diagonal in the flat bands and that such band-off-diagonal states can naturally reconcile all three key challenges (i–iii). More specifically, we find two distinct band-off-diagonal states: one of them transforms under the trivial representation *A* of the system’s point group *C*_6_ (or one of *A*_1,2_ of *D*_6_ if we set the displacement field to zero) but can nonetheless have symmetry-protected nodal lines, akin to Bogoliubov Fermi surfaces discussed in refs. ^56,57^, see Fig. 1a–c for an intuitive visual explanation. The surprising possibility of the existence of such Bogliubov Fermi surfaces without an external magnetic field is unique to twisted graphene systems in that it follows as a direct consequence of both the symmetry and relative flatness of their normal-state bands. The second off-diagonal state transforms under a two-dimensional IR (*E*_2_ of *C*_6_). Its associated chiral state, *E*_2_(1, *i*), which is favored in the mean-field over the nematic one, has the unique property of exhibiting nodal lines or being fully gapped depending on the filling fraction, even when the order parameter is kept fixed. We supplement our general symmetry arguments and phenomenological models with Hartree-Fock (HF) calculations on the continuum model, studying a variety of different pairing mechanisms. We find that nodal band-off-diagonal pairing is favored by the optical *A*_1_ and *B*_1_ phonon modes and by fluctuations of a time-reversal symmetric intervalley coherent (T-IVC) state (the T-IVC state has Kekulé order on the graphene scale^58–60^). Evidence for the former has been provided by a recent photoemission study^61^ while evidence for the latter comes from recent STM experiments^7^. Furthermore, also fluctuations of a time-reversal-symmetric sublattice polarized state (SLP+) are attractive in the band-off-diagonal channel (see Table 2 for a formal definition of the order parameters). We also show that fluctuations of both T-IVC and of a nematic, time-reversal symmetric IVC order^62^ favor either the band-off-diagonal *A* or an *E*_1_ state with band-diagonal components, which may also be nodal; the winner is determined by the relative amount of nematic IVC and T-IVC fluctuations.

## Results

### Possible pairing states

Let us begin by classifying the superconducting instabilities in graphene moiré systems in the limit where the low-energy bands are spin-polarized but allow for multiple bands. We denote the spinless low-energy fermionic creation operators by ${c}_{{{{{{{{\boldsymbol{k}}}}}}}},\alpha,\eta }^{{{{\dagger}}} }$ with momentum ***k*** in valley *η* = ± , and of band index *α* labeling the upper (*α* = + ) and lower (*α* = − ) quasi-flat bands. As a result of two-fold rotational symmetry, *C*_2*z*_, along the out-of-plane (*z*) direction or effective spinless time-reversal symmetry, Θ, the non-interacting band structure *ξ*_***k***,*α*,*η*_ obeys *ξ*_***k***,*α*,*η*_ = *ξ*_−***k***,*α*,−*η*_ ≡ *ξ*_*η*⋅***k***,*α*_ and intervalley pairing is expected to dominate. A general pairing order parameter in the inter-valley channel couples as

1 $${{{{{{{{\mathcal{H}}}}}}}}}_{{{{{{{{\rm{p}}}}}}}}}=\mathop{\sum}\limits_{{{{{{{{\boldsymbol{k}}}}}}}},\eta= \pm ,\alpha,{\alpha }^{{\prime} }}{c}_{{{{{{{{\boldsymbol{k}}}}}}}},\alpha,\eta }^{{{{\dagger}}} }{\left({{{\Delta }}}_{{{{{{{{\boldsymbol{k}}}}}}}},\eta }\right)}_{\alpha,{\alpha }^{{\prime} }}{c}_{-{{{{{{{\boldsymbol{k}}}}}}}},{\alpha }^{{\prime} },-\eta }^{{{{\dagger}}} }+\,{{\mbox{H.c.}}}\,,$$

where the order parameter ${{{\Delta }}}_{{{{{{{{\boldsymbol{k}}}}}}}},\eta }=-{{{\Delta }}}_{-{{{{{{{\boldsymbol{k}}}}}}}},-\eta }^{T}$ is a matrix in band space. The physical spin texture of the superconductor is entirely determined by the form of the underlying normal-state’s polarization: if the spins are aligned in the two valleys, the superconductor is a non-unitary triplet, while anti-alignment^24,28^ leads to a singlet-triplet admixed state^13,27,28^. In both cases, all of the following states are well defined, with the aforementioned spin structures and symmetries given by appropriate combinations of spinless operations and spin rotations (see Supplementary Appendix A1).

We will classify the pairing states according to the irreducible representations (IRs) of the system’s point group *D*_6_, which is generated by six-fold rotations (*C*_6*z*_) along the *z* axis and two-fold rotation symmetry (*C*_2*x*_) along the in-plane *x* axis. Note a displacement field (*D*_0_ ≠ 0) breaks the in-plane rotations leading to the point group *C*_6_. Importantly, all IRs of *D*_6_ and *C*_6_ are either even or odd under *C*_2*z*_. Choosing the phases of the Bloch states such that *C*_2*z*_ acts as *c*_***k***,*α*,*η*_ → *c*_−***k***,*α*,−*η*_, it holds

2 $${C}_{2z}:\quad {{{\Delta }}}_{{{{{{{{\boldsymbol{k}}}}}}}},\eta }\quad \longrightarrow \quad {{{\Delta }}}_{-{{{{{{{\boldsymbol{k}}}}}}}},-\eta }=-{{{\Delta }}}_{{{{{{{{\boldsymbol{k}}}}}}}},\eta }^{T}.$$

This immediately implies that the pairing states in all IRs even under *C*_2*z*_ (*A*_1_, *A*_2_, *E*_2_ of *D*_6_) must be anti-symmetric in band space and, thus, entirely band-off-diagonal, whereas the order parameters of the other IRs (*B*_1_, *B*_2_, *E*_1_) are symmetric and can contain both band-diagonal and band-off-diagonal components. While superconducting order parameters with finite band-off-diagonal components are rather common in multi-band systems, the existence of pairing states that are constrained to be entirely band-off-diagonal is rather unique and follows from the combination of *C*_2*z*_ symmetry and the spin polarization in the normal state. Importantly, this is unaffected by strain or nematic order breaking *C*_3*z*_ as long as *C*_2*z*_ remains, which guarantees that there are IRs with entirely band-off-diagonal order parameters.

Choosing the phase conventions of the Bloch states such that *C*_2*x*_ and *C*_3*z*_ act as ${c}_{{{{{{{{\boldsymbol{k}}}}}}}},\alpha,\eta }\to {({\sigma }_{z})}_{\alpha \alpha }{c}_{({k}_{x},-{k}_{y}),\alpha,\eta }$ and ${c}_{{{{{{{{\boldsymbol{k}}}}}}}},\alpha,\eta }\to {c}_{{C}_{3z}{{{{{{{\boldsymbol{k}}}}}}}},\alpha,\eta }$, respectively, the resulting candidate order parameters are summarized in Table 1. Note that a momentum-independent representation of *C*_2*x*_ must be *σ*_*z*_ due to the bands’ eigenvalues at the Γ-M line, which in turn are connected to the topological obstruction of the flat bands^63^. The reality (Hermiticity) constraint in Table 1 on *χ*, *X*, and *Y* ($\hat{\chi },\, \hat{X}$, and $\hat{Y}$) comes from the residual spinless time-reversal symmetry Θ of the normal state^64,65^. The two two-dimensional IRs *E*_1,2_ are each associated with three pairing states—two nematic phases *E*_1,2_(1, 0), *E*_1,2_(0, 1) and one chiral state *E*_1,2_(1, *i*).

**Table 1:** Summary of pairing states in spin-polarized flat bands

IR of *D*_6_	${{{\Delta }}}_{{{{{{{{\boldsymbol{k}}}}}}}},\eta }=-{{{\Delta }}}_{-{{{{{{{\boldsymbol{k}}}}}}}},-\eta }^{T}$	nodes	IR of *C*_6_
*A*_1_	${\sigma }_{y}{\chi }_{\eta \cdot {{{{{{{\boldsymbol{k}}}}}}}}},{\chi }_{{C}_{2x}{{{{{{{\boldsymbol{k}}}}}}}}}=-{\chi }_{{{{{{{{\boldsymbol{k}}}}}}}}}$	ln/pt or ln	*A*
*A*_2_	${\sigma }_{y}{\chi }_{\eta \cdot {{{{{{{\boldsymbol{k}}}}}}}}},{\chi }_{{C}_{2x}{{{{{{{\boldsymbol{k}}}}}}}}}={\chi }_{{{{{{{{\boldsymbol{k}}}}}}}}}$	ln/n	*A*
*E*_2_(1, 0)	*σ*_*y*_*Y*_*η*⋅***k***_	ln/ln or pt	*E*_2_(1, 0)
*E*_2_(0, 1)	*σ*_*y*_*X*_*η*⋅***k***_	ln/ln or pt	*E*_2_(1, 0)
*E*_2_(1, *i*)	${\sigma }_{y}({X}_{\eta \cdot {{{{{{{\boldsymbol{k}}}}}}}}}+i\,{Y}_{\eta \cdot {{{{{{{\boldsymbol{k}}}}}}}}})$	ln/ln or n	*E*_2_(1, *i*)
*B*_1_	$\eta {\hat{\chi }}_{\eta \cdot {{{{{{{\boldsymbol{k}}}}}}}}},{\sigma }_{z}{\hat{\chi }}_{{C}_{2x}{{{{{{{\boldsymbol{k}}}}}}}}}{\sigma }_{z}={\hat{\chi }}_{{{{{{{{\boldsymbol{k}}}}}}}}}$	n	*B*
*B*_2_	$\eta {\hat{\chi }}_{\eta \cdot {{{{{{{\boldsymbol{k}}}}}}}}},{\sigma }_{z}{\hat{\chi }}_{{C}_{2x}{{{{{{{\boldsymbol{k}}}}}}}}}{\sigma }_{z}=-{\hat{\chi }}_{{{{{{{{\boldsymbol{k}}}}}}}}}$	pt	*B*
*E*_1_(1, 0)	$\eta {\hat{X}}_{\eta \cdot {{{{{{{\boldsymbol{k}}}}}}}}}$	pt	*E*_1_(1, 0)
*E*_1_(0, 1)	$\eta {\hat{Y}}_{\eta \cdot {{{{{{{\boldsymbol{k}}}}}}}}}$	pt	*E*_1_(1, 0)
*E*_1_(1, *i*)	$\eta ({\hat{X}}_{\eta \cdot {{{{{{{\boldsymbol{k}}}}}}}}}+i{\hat{Y}}_{\eta \cdot {{{{{{{\boldsymbol{k}}}}}}}}})$	n	*E*_1_(1, *i*)

Here *χ*_***k***_ (${\hat{\chi }}_{{{{{{{{\boldsymbol{k}}}}}}}}}$) is a real-valued (real and symmetric 2 × 2 matrix-valued) MBZ-periodic function invariant under *C*_3*z*_. Furthermore, *X*_***k***_ and *Y*_***k***_ (${\hat{X}}_{{{{{{{{\boldsymbol{k}}}}}}}}}$ and ${\hat{Y}}_{{{{{{{{\boldsymbol{k}}}}}}}}}$) transform as *x* and *y* under *D*_3_, generated by *C*_3*z*_ and *C*_2*x*_, while also being real (and symmetric). The third column indicates the type of nodes—line (ln), point (pt), or none (n)—on a generic Fermi surface for sufficiently small/large order-parameter magnitudes; options separated by “or” indicates that this depends on the normal-state band splitting, see main text. The last column shows which states merge when *D*_0_ ≠ 0, reducing the point group from *D*_6_ to *C*_6_.

### Spectral properties

We here have the rather unique situation that there are pairing channels, associated with the IRs *A*_1,2_ and *E*_2_, where the pairing is constrained by *C*_2*z*_ to be entirely band-off-diagonal. One immediate very unusual consequence is that the superconducting order-parameter transforming under the trivial representation (*A*_1_) has a symmetry-imposed line of zeros along the Γ-M line, and hence a nodal point in the spectrum. This is related to the topology-induced non-trivial representation of *C*_2*x*_ in band space. We refer to ref. ^39^ for the discussion of other topological nodal points for pairing in obstructed TBG bands. As we will show next, band-off-diagonal pairing leads to additional unusual spectral properties with far-reaching consequences for graphene moiré systems. To this end, consider the following effective Hamiltonian, ${{{{{{{{\mathcal{H}}}}}}}}}_{{\sigma }_{y}}={\sum }_{{{{{{{{\boldsymbol{k}}}}}}}}}{c}_{{{{{{{{\boldsymbol{k}}}}}}}},\alpha,\eta }^{{{{\dagger}}} }{c}_{{{{{{{{\boldsymbol{k}}}}}}}},\alpha,\eta }{\xi }_{\eta \cdot {{{{{{{\boldsymbol{k}}}}}}}},\alpha }+{\sum }_{{{{{{{{\boldsymbol{k}}}}}}}}}[{{{\Delta }}}_{{{{{{{{\boldsymbol{k}}}}}}}}}\,{c}_{{{{{{{{\boldsymbol{k}}}}}}}},+}^{{{{\dagger}}} }{\sigma }_{y}{c}_{-{{{{{{{\boldsymbol{k}}}}}}}},-}^{{{{\dagger}}} }+\,{{\mbox{H.c.}}}\,]$, where the scalar function Δ_***k***_ describes the form of pairing. We will here study two cases that are conventionally considered to be fully gapped, (i) a momentum-independent “*s*-wave state” (*A*_2_ or *A* pairing in Table 1) where Δ_***k***_ = Δ_0_ and (ii) a “chiral *p*-wave” state, or more precisely an *E*_2_(1, *i*) state, where Δ_***k***_ = Δ_0_(*X*_***k***_ + *iY*_***k***_) with (*X*_***k***_, *Y*_***k***_) being smooth, MBZ-periodic functions transforming as (*x*, *y*) under *C*_3*z*_. Furthermore, we parameterize the dispersion, *ξ*_*η*⋅***k***,*α*_, of the two flat bands (*α* = ± ) in valley *η* = ± as *ξ*_***k***,*α*_ = *ϵ*_***k***_ − *μ* + *α* *δ*_***k***_, where *ϵ*_***k***_ and *δ*_***k***_ are *C*_3*z*_ (and, for *D*_0_ = 0, *C*_2*x*_) symmetric functions.

The Bogoliubov spectrum of ${{{{{{{{\mathcal{H}}}}}}}}}_{{\sigma }_{y}}$ has four bands, given by $\pm{\delta }_{{{{{{{{\boldsymbol{k}}}}}}}}}\pm \sqrt{{({\epsilon }_{{{{{{{{\boldsymbol{k}}}}}}}}}-\mu )}^{2}+| {{{\Delta }}}_{{{{{{{{\boldsymbol{k}}}}}}}}}{| }^{2}}$. Consequently, the excitation gap at momentum ***k*** reads as

3 $${{\Delta }}{E}_{{{{{{{{\boldsymbol{k}}}}}}}}}=\Big | | {\delta }_{{{{{{{{\boldsymbol{k}}}}}}}}}| -\sqrt{{({\epsilon }_{{{{{{{{\boldsymbol{k}}}}}}}}}-\mu )}^{2}+| {{{\Delta }}}_{{{{{{{{\boldsymbol{k}}}}}}}}}{| }^{2}}\Big|,$$

which is shown in Fig. 1d, and therefore exhibits nodes where $| {\delta }_{{{{{{{{\boldsymbol{k}}}}}}}}}|=\sqrt{{({\epsilon }_{{{{{{{{\boldsymbol{k}}}}}}}}}-\mu )}^{2}+| {{{\Delta }}}_{{{{{{{{\boldsymbol{k}}}}}}}}}{| }^{2}}$. As long as the band structure has Dirac points, there are points ***k***_*D*_ in the Brillouin zone with ${\delta }_{{{{{{{{{\boldsymbol{k}}}}}}}}}_{D}}=0$, associated with the blue cross in Fig. 1d. Furthermore, for a metallic normal state, *μ* must be within the bandwidth and, hence, there must be a region *R* in momentum space where ∣*δ*_***k***_∣ > ∣*ϵ*_***k***_ − *μ*∣. For the momentum-independent *A*_2_ state, Δ_***k***_ = Δ_0_, this implies that there exists ${{{\Delta }}}_{0}^{c} > 0$ such that there is k^*^ ∈ *R* with parameters (such as the blue circle) above the red solid line in Fig. 1d as long as $| {{{\Delta }}}_{0}| < {{{\Delta }}}_{0}^{c}$. By continuity, this means that there must be a nodal point on any line connecting ***k***_*D*_ and k^*^. Consequently, for *μ* within the bandwidth and ${\delta }_{{{{{{{{{\boldsymbol{k}}}}}}}}}_{D}}=0$ for some ***k***_*D*_, the *A*_2_ will always have a nodal line if ∣Δ_0_∣ is sufficiently small, consistent with the intuitive picture based on the Bogoliubov spectrum in Fig. 1a–c.

We illustrate this further in Fig. 1e using a toy model with ${\delta }_{{{{{{{{\boldsymbol{k}}}}}}}}}=t\left|1+{e}^{i{{{{{{{{\boldsymbol{a}}}}}}}}}_{1}\cdot {{{{{{{\boldsymbol{k}}}}}}}}}+{e}^{-i{{{{{{{{\boldsymbol{a}}}}}}}}}_{2}\cdot {{{{{{{\boldsymbol{k}}}}}}}}}\right|$ and ${\epsilon }_{{{{{{{{\boldsymbol{k}}}}}}}}}={t}^{{\prime} }\mathop{\sum }\nolimits_{j=1}^{3}\cos {{{{{{{{\boldsymbol{a}}}}}}}}}_{j}\cdot {{{{{{{\boldsymbol{k}}}}}}}},\, {{{{{{{{\boldsymbol{a}}}}}}}}}_{j}={[{C}_{3z}]\,}^{j-1}{(\sqrt{3},0)}^{T}$. This leads to the second unexpected conclusion that, for any pairing mechanism, including conventional electron–phonon coupling, the leading instability either has nodal lines in a finite region below *T*_*c*_ or transforms non-trivially under the symmetries of the normal state. For electron–phonon pairing (or pairing mediated by the fluctuations of any time-reversal-symmetric order parameter^52^, such as the T-IVC state) this is particularly unexpected since it is generally believed to always lead to a fully gapped state that transforms trivially under all symmetries. In fact, this can be proven in general terms^51,52^, even for spin-orbit-split Fermi surfaces and beyond mean-field theory^52^. The crucial difference to these works, however, is that spinfull time-reversal is broken in our case such that the Fermi-Dirac constraint is inconsistent with a non-sign-changing, band-diagonal pairing state. This leads to the unique situation that although electron–phonon coupling will lead to entirely attractive interactions in the Cooper channel, the superconducting energetics is frustrated: the dominant pairing state is determined by whether the energetic loss due to non-resonant band-off-diagonal Cooper pairs (*A*_2_ pairing) or the costs from sign changes of the order parameter (such as *B*_1_) are less harmful. We will demonstrate this explicitly by a model calculation in sec. “electron–phonon coupling below, where either *A*_2_ or *B*_1_ is dominant, depending on the form of the electron–phonon coupling”.

Let us first, however, discuss the general spectral properties of the “chiral *p*-wave” state which is canonically expected to be fully gapped as long as the Fermi surfaces do not cross the zeros of *X*_***k***_ + *iY*_***k***_. Three of these zeros have to be at the Γ, *K*, and ${K}^{{\prime} }$ points as a consequence of *C*_3*z*_ symmetry. In the absence of fine-tuning, *X*_***k***_ + *iY*_***k***_ will have vortices at these points with vorticity *v* = + 1. As can be seen in Fig. 1f, where we show the phase of *X*_***k***_ + *iY*_***k***_ using an admixture of the two lowest-order terms, the net vorticity of + 3 at these high-symmetry points has to be compensated by anti-vortices at generic momenta. The lowest possible number is three *C*_3*z*_-related vortices, which appear near the M points in Fig. 1f. If it holds ∣*δ*_***k***_∣ > ∣*ϵ*_***k***_ − *μ*∣ at any of these zeros ***k*** = ***k***_*j*_, we obtain a point above the red line in Fig. 1d and, thus, a nodal point along any contour between that ***k***_*j*_ and ***k***_*D*_; as opposed to the *A*_2_ state, this holds irrespective of the value of Δ_0_ and therefore all the way to zero temperature. In summary, we find that also the *E*_2_(1, *i*) “chiral *p*-wave” state is not generically fully gapped but instead will exhibit a nodal line encircling any zero ***k***_*j*_ of *X*_***k***_ + *iY*_***k***_ with $| {\delta }_{{{{{{{{{\boldsymbol{k}}}}}}}}}_{j}}| > | {\epsilon }_{{{{{{{{{\boldsymbol{k}}}}}}}}}_{j}}-\mu |$. This leads to an interesting filling dependence of the superconducting gap, as we illustrate in our toy model in Fig. 1g along with the criterion ${D}_{{{{{{{{{\boldsymbol{k}}}}}}}}}_{j}}:=| {\delta }_{{{{{{{{{\boldsymbol{k}}}}}}}}}_{j}}| -| {\epsilon }_{{{{{{{{{\boldsymbol{k}}}}}}}}}_{j}}-\mu | > 0$ evaluated at the vortices at Γ, K/K’, and near M. Depending on *μ*, *D*_***k***_ is positive only near the Γ point or only in a region surrounding the vortices close to the M points, leading to nodal lines encircling Γ and near the M points, respectively, as shown in the inset of Fig. 1g. These regimes are separated by a fully gapped region where *D*_***k***_ < 0 for all ***k***, which could explain the fully gapped to nodal transition seen in tunneling experiments^49^ when the filling fraction is changed. Note that ${D}_{{{{{{{{{\boldsymbol{k}}}}}}}}}_{j}}=-| {\epsilon }_{{{{{{{{{\boldsymbol{k}}}}}}}}}_{j}}-\mu | \le 0$ for ***k***_*j*_ at the K and K’ points. In Fig. 1g, ${D}_{K}={D}_{{K}^{{\prime} }}$ vanishes close to the top of the band, which simply means that the Fermi surfaces cross the K, K’ points and the superconductor has nodal points for this fine-tuned value of the chemical potential.

### Fluctuation-induced pairing

Having discussed the unique energetics of pairing and spectral properties of the resulting superconductors in spin-polarized quasi-flat-bands with Dirac cones on a general level, we next study these aspects more explicitly by solving the superconducting self-consistency equations in the flat bands common to alternating-twist graphene systems. We will start with pairing induced by fluctuations of a nearby symmetry-broken phase. To this end, we will couple the low-energy electrons introduced in Eq. (1) to a collective bosonic field ${\phi }_{j}({{{{{{{\boldsymbol{q}}}}}}}})={\phi }_{j}^{{{{\dagger}}} }(-{{{{{{{\boldsymbol{q}}}}}}}})$ via

4 $${{{{{{{{\mathcal{H}}}}}}}}}_{\phi }=\mathop{\sum}\limits_{{{{{{{{\boldsymbol{k}}}}}}}},{{{{{{{\boldsymbol{q}}}}}}}},j}{c}_{{{{{{{{\boldsymbol{k}}}}}}}}+{{{{{{{\boldsymbol{q}}}}}}}},\alpha,\eta }^{{{{\dagger}}} }{\lambda }_{\alpha,\eta ;{\alpha }^{{\prime} },{\eta }^{{\prime} }}^{j}{c}_{{{{{{{{\boldsymbol{k}}}}}}}},{\alpha }^{{\prime} },{\eta }^{{\prime} }}{\phi }_{j}({{{{{{{\boldsymbol{q}}}}}}}}),$$

where the Hermitian matrices *λ*^*j*^ capture the nature of the correlated insulating phase; we here choose and normalize *λ*^*j*^ such that ${({\lambda }^{j})}^{2}={\mathbb{1}}$. Both for twisted bi-^9^ and trilayer graphene^14,15,29^, the stable phases emerging out of the *U*(4) × *U*(4)^9^ manifold in the chiral-flat (decoupled) limit are natural candidates. Integrating out the bosonic modes, we obtain an effective electronic interaction which in the for superconductivity relevant intervalley Cooper channel reads as

5 $${{{{{{{{\mathcal{H}}}}}}}}}_{{{{{{{{\rm{int}}}}}}}}}^{\phi }=	 -\mathop{\sum}\limits_{{{{{{{{\boldsymbol{k}}}}}}}},{{{{{{{{\boldsymbol{k}}}}}}}}}^{{\prime} }}{\chi }_{{{{{{{{\boldsymbol{k}}}}}}}}-{{{{{{{{\boldsymbol{k}}}}}}}}}^{{\prime} }}{{{{{{{{\mathcal{V}}}}}}}}}_{(\eta,\alpha,\beta ),({\eta }^{{\prime} },{\alpha }^{{\prime} },{\beta }^{{\prime} })}\\ 	 \times {c}_{-{{{{{{{\boldsymbol{k}}}}}}}},\beta,-\eta }^{{{{\dagger}}} }{c}_{{{{{{{{\boldsymbol{k}}}}}}}},\alpha,\eta }^{{{{\dagger}}} }{c}_{{{{{{{{{\boldsymbol{k}}}}}}}}}^{{\prime} },{\alpha }^{{\prime} },{\eta }^{{\prime} }}{c}_{-{{{{{{{{\boldsymbol{k}}}}}}}}}^{{\prime} },{\beta }^{{\prime} },-{\eta }^{{\prime} }},$$

with vertex

6 $${{{{{{{{\mathcal{V}}}}}}}}}_{(\eta,\alpha,\beta ),({\eta }^{{\prime} },{\alpha }^{{\prime} },{\beta }^{{\prime} })}={t}_{\phi }\mathop{\sum}\limits_{j}{\left[{\lambda }_{\beta,\eta ;{\beta }^{{\prime} },{\eta }^{{\prime} }}^{j}\right]}^{*}{\lambda }_{\alpha,\eta ;{\alpha }^{{\prime} },{\eta }^{{\prime} }}^{j},$$

*t*_*ϕ*_ = ± 1 encoding whether the order parameter is even or odd under time-reversal, Θ*ϕ*_*j*_(***q***)Θ^†^ = *t*_*ϕ*_*ϕ*_*j*_(***q***), and *χ*_***q***_ > 0 denoting the (static) susceptibility of *ϕ*_*j*_.

Before discussing numerical results for the full model, we first focus on perfectly flat bands. In this limit, the leading superconducting instability within mean-field theory is given by the largest eigenvalue of ${{{{{{{\mathcal{V}}}}}}}}$ in Eq. (6) viewed as a matrix in the multi-index (*η*, *α*, *β*). Furthermore, if there is an anti-symmetric, valley-off-diagonal matrix *D* obeying (see Methods)

7 $${[D{\eta }_{x},{\lambda \,}^{j}]}_{-{t}_{\phi }}\equiv D{\eta }_{x}{\lambda }^{j}-{t}_{\phi }{\lambda }^{j}D{\eta }_{x}=0,$$

the associated leading superconducting order parameter in Eq. (1) is given by ${({{{\Delta }}}_{{{{{{{{\boldsymbol{k}}}}}}}},\eta })}_{\alpha,{\alpha }^{{\prime} }}={\delta }_{{{{{{{{\boldsymbol{k}}}}}}}}}{(D{\eta }_{x})}_{\alpha,\eta ;{\alpha }^{{\prime} }\eta }$ with *δ*_***k***_ > 0; here *η*_*j*_ denotes Pauli matrices in valley space and the precise form of *δ*_***k***_ is determined by *χ*(***q***).

### T-IVC fluctuations

Motivated by recent experiments^7^ providing direct evidence for T-IVC order, we start with T-IVC fluctuations as a pairing glue. In the *U*(4) × *U*(4) symmetric limit, the T-IVC state is associated with *λ*^*j*^ = *σ*_0_*η*_*j*_, *j* = *x*, *y*, within our conventions. Since *t*_*ϕ*_ = + 1, we are looking for *Dη*_*x*_ that commutes with *λ*^*j*^. Interestingly, there is a unique anti-symmetric, valley-off-diagonal matrix *D* ∝ *σ*_*y*_*η*_*x*_ with that property, implying that the leading pairing state has the form Δ_***k***,*η*_ = *σ*_*y*_*δ*_***k***_, *δ*_***k***_ > 0. This is exactly the *A*_2_ state in Table 1, which, as discussed above, will have nodal lines at least in the vicinity of *T*_*c*_ when a finite band dispersion is taken into account. Intuitively, the fact that *A*_2_ pairing is favored can be understood by noticing that the valley-off-diagonal form of *λ*^*j*^ leads to an attractive interaction across the valleys, which penalizes the *B*_1_ state with its sign change between the two valleys. In fact, it holds ${{{{{{{{\mathcal{V}}}}}}}}}_{(\eta,\alpha,\beta ),({\eta }^{{\prime} },{\alpha }^{{\prime} },{\beta }^{{\prime} })}=(1-\eta \,{\eta }^{{\prime} })\mathop{\sum }\nolimits_{\mu=0}^{3}{({\sigma }_{\mu }^{*})}_{\alpha,\beta }{({\sigma }_{\mu })}_{{\alpha }^{{\prime} },{\beta }^{{\prime} }}$ showing explicitly that it is repulsive (attractive) in the *B*_1_ (*A*_2_) channel.

**Fig. 2:**
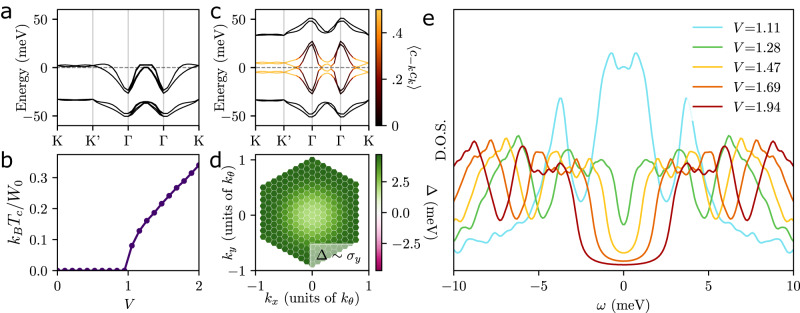
Pairing mediated by T-IVC fluctuations. We show **a** the band structure of the normal state with spin polarization (K, K', and Γ label the high-symmetry points of the moiré scale Brillouin zone) and **b** the critical temperature *T*_*c*_ (in units of the maximum band splitting *W*_0_ ≃ 9.4 meV) as a function of coupling strength *V* measured in units of the critical coupling *V*_*c*,1_ = 105 meV ⋅ nm^2^ obtained from the linearized gap equation. The band structure (with color indicating the band-projected value of the anomalous correlator) of the *A*_2_ state and its order parameter are shown in **c** and **d**. The DOS of the *T* = 0 superconductor for several different values of coupling strength *V* is plotted in **e**. The DOS was computed as ${\sum }_{{{{{{{{\boldsymbol{k}}}}}}}}}\delta \left({E}_{{{{{{{{\boldsymbol{k}}}}}}}}}-\omega \right)$, replacing the *δ* function with Lorentzians with half width at half max 0.3 meV (much smaller than the typical superconducting order parameter). The critical coupling *V*_*c*,2_ where the nodal lines disappear is *V*_*c*,2_ ≃ 1.4*V*_*c*,1_.

To go beyond the flat-band limit, we solve the superconducting mean-field equations numerically. We take the flat TBG bands from the continuum model^66^ as the starting point. To capture the spin-polarized normal state, we supplement it with Coulomb repulsion and perform HF calculation (see Supplementary Appendix A for details). As can be seen in the resulting band structure shown in Fig. 2a with interaction renormalization assuming filling fraction *ν* = 2, this not only pushes one spin flavor below the Fermi level but also induces significant band renormalizations. For our subsequent study of superconducticity, we project onto the two bands at the Fermi level and associate them with the creation operators *c*_***k***,*α*_ in the interactions in Eqs. (4) and (5). In our numerical computations, we choose $\chi ({{{{{{{\boldsymbol{q}}}}}}}})=\frac{1}{{A}_{m}}\frac{V}{{\alpha }^{2}+| {{{{{{{\boldsymbol{q}}}}}}}}{| }^{2}/{k}_{\theta }^{2}}$ where *A*_*m*_ is the real space area of a moiré unit cell, and take *α* = 0.05 for concreteness, although we checked our main conclusion do not crucially depend on this form. In all of our numerics, we work at doping *ν* = 2.5.

As expected, we indeed find that the *A*_2_ state dominates, both right at the critical temperature *T*_*c*_, obtained from the linearized gap equation, and at *T* = 0 as we show by iteratively solving the full self-consistency equation (see Supplementary Appendix C). One crucial effect of the finite dispersion and splitting between the bands is that a finite interaction strength, *V* > *V*_*c*,1_, is required to stabilize the superconducting phase, as can be seen in the plot of *T*_*c*_ in Fig. 2b. Superconductivity ceases to be a weak-coupling instability as the Bloch states (***k***, *α*, *η*) and $(-{{{{{{{\boldsymbol{k}}}}}}}},\, {\alpha }^{{\prime} },-\eta )$ are not degenerate for $\alpha \, \ne \, {\alpha }^{{\prime} }$, cutting off the logarithmic divergence known from BCS theory. The quasi-particle spectrum and order parameter of superconductivity from *T* = 0 numerics are shown in Fig. 2c, d. In accordance with our general discussion above, we observe that the order parameter only has finite components proportional to *σ*_*y*_, which do not mix with the band-even contributions ∝ *σ*_0,*x*,*z*_ as a result of *C*_2*z*_ symmetry. Furthermore, it does not change sign as a function of ***k*** and, for sufficiently small *V* but still with *V* > *V*_*c*,1_, the nodal lines in the superconducting spectrum persist all the way to *T* = 0, while the nodal line is gapped out at low *T* < *T*_*c*_ if *V* > *V*_*c*,2_.

The interaction-strength-dependence of the superconducting gap can be more clearly seen in Fig. 2e, where we show the DOS for the self-consistent solution at *T* = 0. For large *V*, the superconductor becomes fully gapped at *T* = 0, leading to a *U*-shaped DOS. With smaller *V*, the magnitude of the order-parameter decreases and the superconductor eventually exhibits nodal lines, as explained above. In the regime just before these nodal lines appear, there is an increase in the DOS near the Fermi level, roughly when the order parameter and the maximal band splitting are comparable, leading to a V-shaped DOS (green line). The lifetime parameter used to compute the DOS is 0.3 meV; this choice was based on our *k*-grid spacing. While it is not necessarily small with respect to the tunneling gap (which vanishes at *V*_*c*,2_), it is small with respect to Δ(***k***), which is of order 5 meV just as the state is becoming fully gapped for our choice of normal state. This behavior of the DOS with interaction strength may offer a natural explanation for the *U*-shaped tunneling conductance measurements near *ν* = 2 and V-shaped tunneling conductance measurements near *ν* = 3 observed in TTG^49^; if we are considering T-IVC fluctuations of the insulator at *ν* = 2, then it may be reasonable to expect the coupling to these fluctuations could grow weaker as we dope towards *ν* = 3, in line with the experimentally observed *ν* dependence.

Note that the regime we call V-shaped here is strictly speaking fully gapped. However, the crucial difference to the BCS state is that the gap is much smaller than the order-parameter magnitude as a result of the different Bogoliubov spectrum in Eq. (3). This is why, depending not only on the magnitude of the pairing but also on the precise form of the normal state, the resulting tunneling spectra can resemble those observed experimentally^48,49^, such as the green curve in Fig. 2e, making the *A*_2_ state an attractive candidate. The regime of small *V* where stable superconductivity with true Bogoliubov Fermi surfaces is observed can further exhibit a peak at *ω* = 0 which is due to a Van Hove singularity crossing the Fermi level, see blue curve in Fig. 2e; while this peak has not been observed experimentally, its presence crucially depends on details of the normal-state band structure and is only found to be energetically favored in a very small regime of *V* in our model.

### Electron–phonon coupling

To illustrate that the off-diagonal *A*_2_ state is more generally favored beyond just T-IVC fluctuations, we next discuss electron–phonon coupling, which is frequently considered a plausible pairing mechanism for twisted moiré systems^33,35–39^. Similar to ref. ^35^, we use that the optical *A*_1_, *B*_1_, and *E*_2_ phonon modes are known^67^ to dominate the electron–phonon coupling in single-layer graphene. As these are optical phonons, we further assume that the impact of the interlayer coupling on the phonons can be neglected and arrive at

8 $${{{{{{{{\mathcal{H}}}}}}}}}_{EP}=	 \int\,{{{{{{{\rm{d}}}}}}}}{{{{{{{\boldsymbol{r}}}}}}}}\,{\psi }_{\ell,s}^{{{{\dagger}}} }({{{{{{{\boldsymbol{r}}}}}}}})\left[{g}_{{A}_{1}}{{{\Lambda }}}_{{A}_{1}}{u}_{{A}_{1},\mu }({{{{{{{\boldsymbol{r}}}}}}}})\right. \\ 	 \left.+{g}_{{B}_{1}}{{{\Lambda }}}_{{B}_{1}}{u}_{{B}_{1},\mu }({{{{{{{\boldsymbol{r}}}}}}}})+{g}_{{E}_{2}}{{{{{{{{\boldsymbol{\Lambda }}}}}}}}}_{{E}_{2}}\cdot {{{{{{{{\boldsymbol{u}}}}}}}}}_{{E}_{2},\mu }({{{{{{{\boldsymbol{r}}}}}}}})\right]{({{{{{{{{\boldsymbol{v}}}}}}}}}_{\mu })}_{\ell }{\psi }_{\ell,s}({{{{{{{\boldsymbol{r}}}}}}}})$$

for the electron–phonon coupling, where ***v***_*μ*_ encode the layer structure of the modes (see Methods). Symmetry dictates that the vertices Λ_*g*_ are given by ${{{\Lambda }}}_{{A}_{1}}={\eta }_{x}{\rho }_{x},\, {{{\Lambda }}}_{{B}_{1}}={\eta }_{y}{\rho }_{x}$, and ${{{{{{{{\boldsymbol{\Lambda }}}}}}}}}_{{E}_{2}}=({\eta }_{z}{\rho }_{y},-{\rho }_{x})$ where *ρ* acts on the microscopic sublattice basis. Integrating out the phonons and projecting to the flat bands, we obtain an effective electron-electron interaction (see Methods)

9 $${{{{{{{\mathcal{H}}}}}}}}_{{{{{{{{\rm{int}}}}}}}}}^{C}=	 -\mathop{\sum}\limits_{{{{{{{{\boldsymbol{k}}}}}}}},{{{{{{{{\boldsymbol{k}}}}}}}}}^{{\prime} }}{V}_{g}{\left[{\lambda }_{{{{{{{{\boldsymbol{k}}}}}}}},\beta,\eta ;{{{{{{{{\boldsymbol{k}}}}}}}}}^{{\prime} },{\beta }^{{\prime} },{\eta }^{{\prime} }}^{g,j,\mu }\right]}^{*}{\lambda }_{{{{{{{{\boldsymbol{k}}}}}}}},\alpha,\eta ;{{{{{{{{\boldsymbol{k}}}}}}}}}^{{\prime} },{\alpha }^{{\prime} },{\eta }^{{\prime} }}^{g,j,\mu }\\ 	 \times {c}_{-{{{{{{{\boldsymbol{k}}}}}}}},\beta,-\eta }^{{{{\dagger}}} }{c}_{{{{{{{{\boldsymbol{k}}}}}}}},\alpha,\eta }^{{{{\dagger}}} }{c}_{{{{{{{{{\boldsymbol{k}}}}}}}}}^{{\prime} },{\alpha }^{{\prime} },{\eta }^{{\prime} }}{c}_{-{{{{{{{{\boldsymbol{k}}}}}}}}}^{{\prime} },{\beta }^{{\prime} },-{\eta }^{{\prime} }},$$

where the coupling constants *V*_*g*_ of the three different phonon modes *g* = *A*_1_, *B*_1_, *E*_2_ are estimated to obey ${V}_{{A}_{1}}={V}_{{B}_{1}}\simeq 1.33{V}_{{E}_{2}}$ for parallel spins in the two valleys, while ${V}_{{A}_{1}}={V}_{{B}_{1}}=0$ for anti-parallel spins. From Eq. (9), it is clear that the induced interaction would be always completely attractive if we focused on intra-band pairing, $\alpha={\alpha }^{{\prime} }=\beta={\beta }^{{\prime} }$, which in spinful systems generically favors the trivial pairing channel^51,52^. In our case, the combination of two energetically close bands and the trivial pairing being purely band-off-diagonal leads to competition between different superconductors, even with electron–phonon coupling alone.

**Fig. 3:**
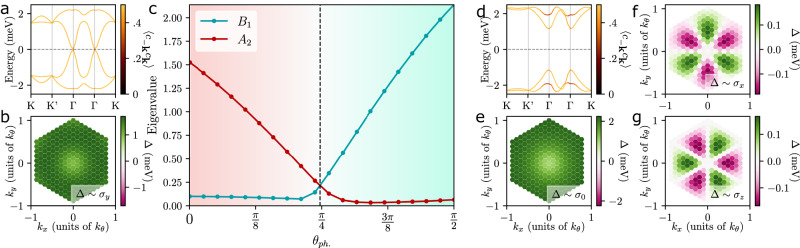
Pairing from electron–phonon coupling. We show **a** the band structure and **b** the self-consistent order parameter of the *A*_2_ pairing for *θ*_ph_ = 0 and *T* = 0. The eigenvalues corresponding to the *A*_2_ and *B*_1_ pairings in the linearized gap equation at *T* = 5 K, which is close to their *T*_*c*_, are shown in **c** as a function of *θ*_ph_. We show an example of the band structure (**d**) of the *B*_1_ pairing and its order parameter (**e**, **f**, **g**). In accordance with symmetry, the *A*_2_ (*B*_1_) state only has order-parameter components ∝ *σ*_*y*_ ( ∝ *σ*_0,*x*,*z*_). We took *ν* = 2.5 and *V*_0_ = 250 meV ⋅ (nm)^2^ with a continuum-model bandwidth ≃ 2 meV. We point out that if *A*_1_ phonons are dominant, as suggested by recent experimental work^61^ and past theoretical study in mono-layer graphene^68^, we would expect our *A*_2_ pairing to dominate assuming the pairing potential is sufficiently large. We also emphasize that although the pairing functions for *A*_2_ pairing (b) when *θ* = 0 and *B*_1_ pairing when *θ* = *π*/2 (**e**, **f**, **g**) are roughly equal, the excitation spectra show the *B*_1_ state with a band gap on the order of the pairing strength (**d**) while the *A*_1_ state’s band gap is nearly zero, see **a**.

To demonstrate this, we study intra-valley pairing within the mean-field approximation and parametrize the relative strength of the different phonon modes with an angle variable *θ*_ph_ according to ${V}_{{A}_{1}}={V}_{{B}_{1}}={V}_{0}\cos {\theta }_{{{{{{{{\rm{ph}}}}}}}}},{V}_{{E}_{2}}={V}_{0}\sin {\theta }_{{{{{{{{\rm{ph}}}}}}}}}$. The results of the mean-field calculation are summarized in Fig. 3. We see that the *A*_2_ pairing state is favored by the intervalley phonons (*θ*_ph_ = 0) inspite of its band-off-diagonal nature leading to a suppressed gap [see Fig. 3a]. This is natural as these phonons mediate an attractive interaction between the two valleys which disfavors the *B*_1_ state, similar to T-IVC fluctuations. In fact, focusing on the leading, momentum-independent term, ${\lambda }_{{{{{{{{\boldsymbol{k}}}}}}}},{{{{{{{{\boldsymbol{k}}}}}}}}}^{{\prime} }}^{g,+}\to {\lambda }^{g,+},g={A}_{1},{B}_{1}$, symmetry dictates ${\lambda }^{{A}_{1},+}\propto {\sigma }_{0}{\eta }_{1}$ and ${\lambda }^{{B}_{1},+}\propto {\sigma }_{0}{\eta }_{2}$ in the chiral limit (see Supplementary Appendix D3). This maps the problem exactly to that of T-IVC fluctuations, immediately explaining why the order parameter has a fixed sign in Fig. 3b. As *θ*_ph_ is increased, the *B*_1_ state is favored (roughly for *θ*_ph_ > *π*/4) as can be seen in Fig. 3c. This is expected since the intra-valley *E*_2_ phonon mediates an attractive interaction within each valley such that the energy gain due to the enhanced gap [Fig. 3d], associated with the band-diagonal matrix elements of the *B*_1_ state, will overcompensate the energetic loss due to the sign change of *B*_1_’s order-parameter between the two valleys. This picture is consistent with the dominant and non-sign-changing nature of the band-diagonal components of the *B*_1_ state, see Fig. 3e–g. Finally, this behavior can also be understood by applying the commutator criterion in Eq. (7) in the microscopic sublattice basis, see Supplementary Appendix D1.

This shows that, as opposed to the conventional scenario^51,52^, there are two possible leading superconducting states and the superconducting pairing state does not transform trivially under the symmetries of the system even when phonons alone provide the pairing glue. We have checked in our *T* = 0 numerics that a 60–70 meV ⋅ (nm)^2^ coupling to *A*_1_ and *B*_1_ phonons (based on ref. ^67^) is roughly of the order needed to stabilize the *A*_2_ pairing, assuming the normal state is the flat bands of the un-renormalized continuum model, which in our case has a bandwidth of 2 meV. However, we note that if the interaction-renormalized band splitting is much larger than the continuum-model bandwidth, or if the normal state has anti-parallel spins in either valley, additional particle-hole fluctuations, such as those of T-IVC order, will also be required for pairing. An interesting scenario arises for anti-parallel spins in the two valleys as a magnetic field will cant the spins and, hence, increase the projection of the intervalley phonon matrix elements to the flat bands. At least in TTG, with the suppressed orbital coupling, this could give rise to re-entrant superconductivity at high fields^25^.

### Other particle-hole fluctuations

**Fig. 4:**
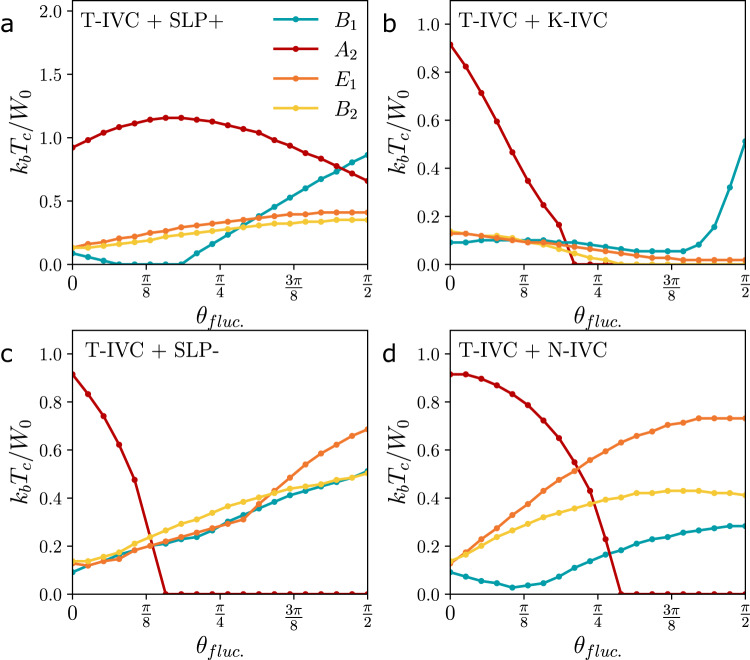
Pairing for different particle-hole fluctuations. These are defined by the coupling matrices ${\bar{\lambda }}^{j}$ listed in Table 2. Similar to Fig. 3c, we show *T*_*c*_ of the leading pairing states, where *θ*_fluc._ tunes the relative strength between T-IVC-induced interactions ($\propto \cos {\theta }_{{{{{{{{\rm{fluc}}}}}}}}.}$) and interactions ($\propto \sin {\theta }_{{{{{{{{\rm{fluc}}}}}}}}.}$) coming from fluctuations of **a** SLP+, **b** K-IVC, **c** SLP−, and **d** N-IVC fluctuations.

Finally, we discuss pairing induced by fluctuations of other particle-hole instabilities. In Table 2, we list the resulting leading superconductors taking *λ*^*j*^ in Eq. (4) to be any of the different strong-coupling candidate order parameters^9,13–15,29^. In particular, in addition to the T-IVC, we will consider the time-reversal-odd Kramers intervalley coherent state (K-IVC), and time-reversal-odd and -even sublattice polarized states (SLP− and SLP+). To analyze how sensitive our conclusions are to the precise form of the coupling of the strong-coupling fluctuating orders to the electrons, we also perform numerics by projecting momentum-independent coupling vertices in the microscopic basis with the correct symmetries (see, e.g., Table II in ref. ^13^), listed as ${\bar{\lambda }}^{j}$ in Table 2, to the flat bands. In the band basis, this leads to momentum-dependent coupling vertices, cf. Eq. (9). Motivated by recent experiments^7^, we will also consider fluctuations of an additional nematic, time-reversal symmetric, layer-odd, intervalley coherent state (N-IVC)^62^ which is not a candidate ground state in the strong-coupling limit; unlike the other strong-coupling ground states, the N-IVC has no momentum-independent representation in the flat-band basis but does have a momentum-independent matrix order parameter in the sublattice basis which takes the form ${\lambda }^{(j,{j}^{{\prime} })}={({\eta }_{x},\, {\eta }_{y})}_{j}{({\rho }_{0},\, {\rho }_{z})}_{{j}^{{\prime} }}$. The results for fluctuations of the projected strong-coupling orders ${\bar{\lambda }}^{j}$ in Table 2 and of the projected N-IVC state are shown in Fig. 4, where we use the angle *θ*_fluc._ to tune the relative strength between T-IVC and any of the other type of fluctuation-induced interactions by multiplying the T-IVC interaction potential with $\cos ({\theta }_{{{{{{{{\rm{fluc}}}}}}}}.})$ and the other fluctuation potential with $\sin ({\theta }_{{{{{{{{\rm{fluc}}}}}}}}.})$. In our microscopic numerics, we have taken a potential form $\chi ({{{{{{{\boldsymbol{q}}}}}}}})=\frac{1}{{A}_{m}}\frac{V}{{\alpha }^{2}+| {{{{{{{\boldsymbol{q}}}}}}}}{| }^{2}/{k}_{\theta }^{2}}$ again with *α* = 0.2 and with *V* = 4200 meV ⋅ (nm)^2^. We chose the value of *V* such that the transitions between the different pairing states are clearly visible in Fig. 4 when varying *θ*_fluc._. In accordance with the prediction for ${\bar{\lambda }}^{j}$ in Table 2, SLP + fluctuations further stabilize the *A*_2_ superconductor, see Fig. 4a. As such, the band-diagonal *B*_1_ superconducting channel, where SLP + fluctuations are also attractive, can become the leading channel (favored over *A*_2_ as a result of the finite bandwidth) only very close to *θ*_fluc._ = *π*/2. K-IVC fluctuations, however, are repulsive for *A*_2_ pairing and favor the *B*_1_ state more strongly.

**Table 2:** Leading superconducting states in the flat-band limit, following from Eq. (7), for pairing mediated by fluctuations of the indicated orders, defined by using *λ*^*j*^ in Eq. (4)

Fluctuating order			Leading superconductor	
type	*λ*^*j*^	${\bar{\lambda }}^{j}$	${{{\Delta }}}_{{{{{{{{\boldsymbol{k}}}}}}}},\eta }^{}$	IR
T-IVC	*σ*_0_*η*_*x*,*y*_	*ρ*_*x*_*η*_*x*,*y*_	*σ*_*y*_*δ*_***k***_	*A*_2_
K-IVC	*σ*_*y*_*η*_*x*,*y*_	*ρ*_*y*_*η*_*x*,*y*_	*σ*_0_*ηδ*_***k***_	*B*_1_
SLP +	*σ*_*y*_*η*_*z*_	*ρ*_*z*_*η*_0_	*σ*_*y*_*δ*_***k***_, *σ*_0_*ηδ*_***k***_	*A*_2_, *B*_1_
SLP −	*σ*_*y*_*η*_0_	*ρ*_*z*_*η*_*z*_	*σ*_*x*_*ηδ*_***k***_, *σ*_*z*_*ηδ*_***k***_	*B*_2_, *B*_1_

Here, *δ*_***k***_ > 0 and states separated by commas are degenerate. The couplings in the microscopic basis, used in Fig. 4 for the respective orders, are listed under ${\bar{\lambda }}^{j}$. Except for SLP−, the leading superconducting states for *λ*^*j*^ and ${\bar{\lambda }}^{j}$ are the same (cf. Fig. 4 and Supplementary Appendix D1).

So far, the strong-coupling (*λ*^*j*^) and sublattice (${\bar{\lambda }}^{j}$) form of the couplings in Table 2 lead to the same conclusions. This is different for SLP− fluctuations (Fig. 4d), where the projection-induced momentum dependence in the band basis can stabilize the *E*_1_ superconductor. This can be understood by applying Eq. (7) in a sublattice basis (see Supplementary Appendix D1). We also find the *E*_1_ state when fluctuations of the N-IVC state of ref. ^62^ dominate. Examples of the *E*_1_ nematic and *B*_2_ order parameters that emerge for SLP− fluctuations or N-IVC fluctuations are shown in Supplementary Appendix F. We point out that the nematic *E*_1_ pairing is also an interesting candidate given that despite having nonzero pairing in the *σ*_0_, *σ*_*x*_, *σ*_*z*_ channels, it will be nodal as long as the *σ*_*x*_ components do not gap out the nodes in the band-diagonal parts.

## Discussion

Taken together, we see that the proposed band-off-diagonal *A*_2_ superconductor is an especially attractive candidate for TBG and TTG: first, it can lead to both V-shaped or *U*-shaped DOS, depending on lifetime parameters, the normal state, and the coupling strength *V*, see Fig. 2e. As these parameters might vary from sample to sample and within a sample (e.g., *V* is expected to decrease upon doping further away from the insulator), this can naturally explain the tunneling data of^48,49^. We emphasize however that at least at the level of our mean-field numerics, we only expect a V-shape in the regime where the superconducting pairing is of the order of the bandwidth; this is the regime, where although the pairing is finite and can be quite large, the gap in the superconducting spectrum is either just closing or very small relative to the pairing. Increasing the pairing further will lead to an evolution from *V* to *U*-shaped while decreasing the pairing will eventually lead to a nodal Fermi surface and presumably a peak at zero energy in the DOS. Second, despite its interband nature, *A*_2_ is the unique pairing state that is favored by fluctuations of two out of the four strong-coupling candidates we consider for the correlated insulator, see Fig. 4a–c. What is more, this includes the T-IVC state, signatures of which are observed in recent experiments^7^. Finally, it is also favored by the likely dominant^61,68^ optical intervalley phonon modes. We emphasize that, both in the case of fluctuating correlated insulators and phonons, the minimum attractive coupling needed to stabilize a purely band-off-diagonal state depends on the energy splitting between the two flat bands in the normal state; if the bands of our normal state are closer to degenerate, irrespective of the total bandwidth, the needed coupling to stabilize the *A*_2_ pairing in mean-field will decrease.

The other band-off-diagonal superconductor we identify transforms under the IR *E*_2_, i.e., can be thought of as a *p*-wave state. Its spectral properties also agree well with the experiment as the chiral configurations, *E*_2_(1, *i*), which is favored within mean-field theory over a nematic *E*_2_ state, can also have nodal regions, depending on filling. As can be seen in Fig. 1g, this can lead to a transition from gapped to nodal when increasing the electron filling starting at *ν* ≃ 2. However, as opposed to the *A*_2_ state, *E*_2_ does not naturally appear as leading instability when considering optical phonons or fluctuations of any of the strong-coupling order parameters of the correlated insulator. While this makes it energetically less natural than *A*_2_, we cannot exclude it since its phenomenology agrees well with the experiment and since the precise form of the coupling of the dominant low-energy collective excitations are not known—significant momentum dependencies beyond *λ*^*j*^ and ${\bar{\lambda }}^{j}$ in Table 2 could stabilize *E*_2_ pairing as well. We also find in our numerics a nematic *E*_1_ state which may be preferred over its chiral version in the presence of sufficient strain or due to fluctuation corrections^27,53–55^. We find the *E*_1_ state is the leading instability of nematic IVC fluctuations and SLP − fluctuations, and is a subleading instability of T-IVC fluctuations. The *E*_1_ state is interesting in its own right, as it can also be nodal.

As superconductivity might further coexist with T-IVC order^7^, we have checked (see Supplementary Appendix E) that this does not alter our main observation: the preserved *C*_2*z*_ symmetry still allows for entirely band-off-diagonal states, with transitions from nodal to full gapped, which are stabilized (among other fluctuations) by intervalley phonons.

For the future, it will be interesting to go beyond mean-field and analyze the competition of our band-off-diagonal states with odd-frequency pairing, which we study in a follow-up work^69^. It also seems promising to study Andreev reflection^48,49^ for our interband pairing scenario. On a more general level, our work shows that the observation of nodal pairing in twisted graphene systems does not immediately exclude a chiral superconducting state nor an entire electron–phonon-based pairing mechanism. It illustrates that a microscopic understanding of the superconducting states in graphene moiré systems requires taking into account their intrinsically multi-band nature.

*Note added*. Just before posting our work, ref. ^70^ appeared online, which discusses pairing induced by *A*_1_ phonons in spinful TBG bands.

## Methods

### Flat-band limit

To derive Eq. (7), we take the flat-band limit, *ξ*_***k***,±_ → 0, in the linearized gap equation. For the interaction defined in Eqs. (4)–(6)), we get (with moiré cell area *A*_*m*_)

10 $${({{{\Delta }}}_{{{{{{{{\boldsymbol{k}}}}}}}},\eta })}_{\beta,{\beta }^{{\prime} }}=	 {t}_{\phi }\frac{1}{4{A}_{m}T}\mathop{\sum}\limits_{{{{{{{{{\boldsymbol{k}}}}}}}}}^{{\prime} }}{\chi }_{{{{{{{{\boldsymbol{k}}}}}}}}-{{{{{{{{\boldsymbol{k}}}}}}}}}^{{\prime} }} \\ 	 \times \mathop{\sum}\limits_{j}{\left[{\lambda }_{{\beta }^{{\prime} },\eta ;{\alpha }^{{\prime} },{\eta }^{{\prime} }}^{j}\right]}^{*}{\lambda }_{\beta,\eta ;\alpha,{\eta }^{{\prime} }}^{j}{({{{\Delta }}}_{{{{{{{{{\boldsymbol{k}}}}}}}}}^{{\prime} },{\eta }^{{\prime} }})}_{\alpha,{\alpha }^{{\prime} }}.$$

We define ${({\hat{{{\Delta }}}}_{{{{{{{{\boldsymbol{k}}}}}}}}})}_{\alpha,\eta ;{\alpha }^{{\prime} },{\eta }^{{\prime} }}:={({{{\Delta }}}_{{{{{{{{\boldsymbol{k}}}}}}}},\eta })}_{\alpha,{\alpha }^{{\prime} }}{\delta }_{\eta,{\eta }^{{\prime} }}$ and note that finding the leading superconducting state according to Eq. (10) is equivalent to determining ${\hat{{{\Delta }}}}_{{{{{{{{\boldsymbol{k}}}}}}}}}$ that maximizes the functional

11 $${{{{{{{\mathcal{F}}}}}}}}[{\hat{{{\Delta }}}}_{{{{{{{{\boldsymbol{k}}}}}}}}}]:=\frac{{\sum }_{{{{{{{{\boldsymbol{k}}}}}}}},{{{{{{{{\boldsymbol{k}}}}}}}}}^{{\prime} },j} \, {\chi }_{{{{{{{{\boldsymbol{k}}}}}}}}-{{{{{{{{\boldsymbol{k}}}}}}}}}^{{\prime} }}\,{t}_{\phi }\,{{\mbox{tr}}}\,[{\lambda }^{j}{\hat{{{\Delta }}}}_{{{{{{{{{\boldsymbol{k}}}}}}}}}^{{\prime} }}{({\lambda }^{j})}^{{{{\dagger}}} }{\hat{{{\Delta }}}}_{{{{{{{{\boldsymbol{k}}}}}}}}}^{{{{\dagger}}} }]}{{\sum }_{{{{{{{{\boldsymbol{k}}}}}}}}}\,{{\mbox{tr}}}\,[{\hat{{{\Delta }}}}_{{{{{{{{\boldsymbol{k}}}}}}}}}^{{{{\dagger}}} }{\hat{{{\Delta }}}}_{{{{{{{{\boldsymbol{k}}}}}}}}}]}.$$

Since ${\chi }_{{{{{{{{\boldsymbol{k}}}}}}}}-{{{{{{{{\boldsymbol{k}}}}}}}}}^{{\prime} }} \, > \, 0$, the maximum value will be reached if we can maximize ${t}_{\phi }\,{{\mbox{tr}}}\,[{\lambda }^{j}{\hat{{{\Delta }}}}_{{{{{{{{{\boldsymbol{k}}}}}}}}}^{{\prime} }}{({\lambda }^{j})}^{{{{\dagger}}} }{\hat{{{\Delta }}}}_{{{{{{{{\boldsymbol{k}}}}}}}}}^{{{{\dagger}}} }]$ for each ${{{{{{{\boldsymbol{k}}}}}}}},\, {{{{{{{{\boldsymbol{k}}}}}}}}}^{{\prime} },\, j$ separately. As the Frobenius inner product ${\left\langle A,B\right\rangle }_{F}=\,{{\mbox{tr}}}\,[{A}^{{{{\dagger}}} }B]$ reaches its maximum (minimum) at fixed $\left\langle A,\, A\right\rangle$ and $\left\langle B,\, B\right\rangle$, if *A* = *cB* with *c* > 0 (*c* < 0), ${t}_{\phi }\,{{\mbox{tr}}}\,[{\lambda }^{j}{\hat{{{\Delta }}}}_{{{{{{{{{\boldsymbol{k}}}}}}}}}^{{\prime} }}{({\lambda }^{j})}^{{{{\dagger}}} }{\hat{{{\Delta }}}}_{{{{{{{{\boldsymbol{k}}}}}}}}}^{{{{\dagger}}} }]$ is maximized if ${\hat{{{\Delta }}}}_{{{{{{{{\boldsymbol{k}}}}}}}}}={t}_{\phi }{c}_{{{{{{{{\boldsymbol{k}}}}}}}},{{{{{{{{\boldsymbol{k}}}}}}}}}^{{\prime} }}{\lambda }^{j}{\hat{{{\Delta }}}}_{{{{{{{{{\boldsymbol{k}}}}}}}}}^{{\prime} }}{({\lambda }^{j})}^{{{{\dagger}}} }$ with ${c}_{{{{{{{{\boldsymbol{k}}}}}}}},{{{{{{{{\boldsymbol{k}}}}}}}}}^{{\prime} }} \, > \, 0$. For the ansatz ${\hat{{{\Delta }}}}_{{{{{{{{\boldsymbol{k}}}}}}}}}={\delta }_{{{{{{{{\boldsymbol{k}}}}}}}}}D{\eta }_{x}$ (and *assuming* for now that *δ*_***k***_ has a fixed sign for all ***k***), this is obeyed if

12 $$D{\eta }_{x}={t}_{\phi }{\lambda }^{j}D{\eta }_{x}{({\lambda }^{j})}^{{{{\dagger}}} },\quad \forall j.$$

We state Eq. (12) as the (anti)commutator condition (7) in the main text [equivalent if ${({\lambda }^{j})}^{2}={\mathbb{1}}$], not only because it highlights the simple algebraic and basis independent nature of the condition but also since it emphasizes the similarities to the generalized Anderson theorem of^71,72^.

If we can find a solution to Eq. (12), we know that the maximum (or at least one of the possibly degenerate maxima) of ${{{{{{{\mathcal{F}}}}}}}}[{\hat{{{\Delta }}}}_{{{{{{{{\boldsymbol{k}}}}}}}}}]$ is of the form of ${\hat{{{\Delta }}}}_{{{{{{{{\boldsymbol{k}}}}}}}}}={\delta }_{{{{{{{{\boldsymbol{k}}}}}}}}}D{\eta }_{x}$ where *δ*_***k***_ is obtained as the maximum of the reduced functional

13 $$\widetilde{{{{{{{{\mathcal{F}}}}}}}}}[{\delta }_{{{{{{{{\boldsymbol{k}}}}}}}}}]:=\frac{{\sum }_{{{{{{{{\boldsymbol{k}}}}}}}},{{{{{{{{\boldsymbol{k}}}}}}}}}^{{\prime} }} \, {\chi }_{{{{{{{{\boldsymbol{k}}}}}}}}-{{{{{{{{\boldsymbol{k}}}}}}}}}^{{\prime} }}{\delta }_{{{{{{{{\boldsymbol{k}}}}}}}}}^{*}{\delta }_{{{{{{{{{\boldsymbol{k}}}}}}}}}^{{\prime} }}^{}}{{\sum }_{{{{{{{{\boldsymbol{k}}}}}}}}}| {\delta }_{{{{{{{{\boldsymbol{k}}}}}}}}}{| }^{2}},$$

or equivalently as the largest eigenvector of ${\chi }_{{{{{{{{\boldsymbol{k}}}}}}}}-{{{{{{{{\boldsymbol{k}}}}}}}}}^{{\prime} }}$ viewed as a matrix in ***k*** and ${{{{{{{{\boldsymbol{k}}}}}}}}}^{{\prime} }$. As ${\chi }_{{{{{{{{\boldsymbol{k}}}}}}}}-{{{{{{{{\boldsymbol{k}}}}}}}}}^{{\prime} }} > 0$ (due to stability), the Perron-Frobenium theorem then immediately implies *δ*_***k***_ > 0, in line with out assumption above and as stated in the main text.

### Electron–phonon coupling

To present more details on the electron–phonon coupling, the associated displacement operators in Eq. (8) can be expressed in terms of canonical bosons, *b*_*g*,*α*,*μ*,***q***_,

14 $${({u}_{g,\mu }({{{{{{{\boldsymbol{r}}}}}}}}))}_{j}=\mathop{\sum}\limits_{{{{{{{{\boldsymbol{q}}}}}}}}}\frac{{b}_{g,j,\mu,{{{{{{{\boldsymbol{q}}}}}}}}}{e}^{i{{{{{{{\boldsymbol{q}}}}}}}}\cdot {{{{{{{\boldsymbol{r}}}}}}}}}+\,{{\mbox{H.c.}}}\,}{\sqrt{2NM{\omega }_{g}({{{{{{{\boldsymbol{q}}}}}}}})}},$$

where *j* refers to the two components for the *E*_2_ phonon (is idle for *A*_1_, *B*_1_), *M* is the carbon mass, and *ω*_*g*_(***q***) is the phonon dispersion, characterizing the phononic part of the Hamiltonian, ${{{{{{{{\mathcal{H}}}}}}}}}_{P}={\sum }_{{{{{{{{\boldsymbol{q}}}}}}}}}{\omega }_{g}({{{{{{{\boldsymbol{q}}}}}}}}){b}_{g,j,\mu,{{{{{{{\boldsymbol{q}}}}}}}}}^{{{{\dagger}}} }{b}_{g,j,\mu,{{{{{{{\boldsymbol{q}}}}}}}}}$.

As for ${({{{{{{{{\boldsymbol{v}}}}}}}}}_{\mu })}_{\ell }$ in Eq. (8), *ℓ* = 1, 2 refers to the physical graphene layer in the case of TBG. One can, in principle, choose any orthonormal basis; we will find it convenient to use the layer-exchange even and odd states, ${{{{{{{{\boldsymbol{v}}}}}}}}}_{\pm }={(1,\pm 1)}^{T}/\sqrt{2}$. For TTG, the situation is more involved (see Supplementary Appendix D2), but our arguments about which phonons are attractive in which pairing channels will hold for both systems.

We project ${{{{{{{{\mathcal{H}}}}}}}}}_{EP}$ in Eq. (8) onto the two flat bands (*α* = ± ) in each valley *η* of the spin-polarized continuum-model, leading to a coupling term similar to Eq. (4) with momentum-dependent coupling matrices, ${\lambda }^{j}\to {\lambda }_{{{{{{{{\boldsymbol{k}}}}}}}},{{{{{{{{\boldsymbol{k}}}}}}}}}^{{\prime} }}^{g,j,\mu }$. Investigating the matrix elements ${\lambda }_{{{{{{{{\boldsymbol{k}}}}}}}},{{{{{{{{\boldsymbol{k}}}}}}}}}^{{\prime} }}^{g,j,\mu }$, we notice that they almost vanish for the layer-odd intervalley (*A*_1_, *B*_1_) phonons, which can be understood as a consequence of chiral and particle-hole symmetry (see Supplementary Appendix D3). The situation is the reverse for the intra-valley (*E*_2_) phonons, where the layer-even matrix elements are numerically small and the layer-odd matrix elements dominate. We therefore focus on layer-even (odd) intervalley (intra-valley) phonon couplings.

Neglecting the momentum dependence in the phonon frequencies and retardation effects, the resulting electron-electron interaction in the inter-valley Cooper channel obtained by integrating out the phonons is given by Eq. (9). Here, ${V}_{g}={g}_{g}^{2}/(2N{\omega }_{g}^{2}) \, > \, 0$ and ${V}_{{A}_{1}}={V}_{{B}_{1}}\simeq 1.33{V}_{{E}_{2}}$ results from ${g}_{{A}_{1}}={g}_{{B}_{1}}\simeq {g}_{{E}_{2}}$ and the phonon frequencies estimated in ref. ^67^. Importantly, this only holds for parallel spins in the two valleys. For anti-parallel spins, the projection of the coupling matrices to the flat bands vanishes for the intervalley phonon modes *A*_1_ and *B*_1_ such that ${V}_{{A}_{1}}={V}_{{B}_{1}}=0$.

### Supplementary information

Supplementary Information

Peer Review File

## Data Availability

The data generated in this study are available in the Zenodo database under the accession code https://zenodo.org/record/8381555and in the figshare repository 10.6084/m9.figshare.23897019.

## Code availability

The codes used to generate the plots are available from the corresponding author on request.

## References

[1] Andrei EY, MacDonald AH (2020). Graphene bilayers with a twist. Nat. Mater..

[2] Balents L, Dean CR, Efetov DK, Young AF (2020). Superconductivity and strong correlations in moiré flat bands. Nat. Phys..

[3] Cao Y (2018). Correlated insulator behaviour at half-filling in magic-angle graphene superlattices. Nature.

[4] Lu X (2019). Superconductors, orbital magnets and correlated states in magic-angle bilayer graphene. Nature.

[5] Sharpe AL (2019). Emergent ferromagnetism near three-quarters filling in twisted bilayer graphene. Science.

[6] Nuckolls KP (2020). Strongly correlated chern insulators in magic-angle twisted bilayer graphene. Nature.

[7] Nuckolls, K. P. et al. Quantum textures of the many-body wavefunctions in magic-angle graphene. (2023). 10.1038/s41586-023-06226-x 37587297

[8] Kang J, Vafek O (2019). Strong coupling phases of partially filled twisted bilayer graphene narrow bands. Phys. Rev. Lett..

[9] Bultinck N (2020). Ground state and hidden symmetry of magic-angle graphene at even integer filling. Phys. Rev. X.

[10] Soejima, T., Parker, D. E., Bultinck, N., Hauschild, J., and Zaletel, M. P. Efficient simulation of moiré materials using the density matrix renormalization group. *Phys. Rev. B* **102** 205111 (2020).

[11] Xie, F. et al. Twisted bilayer graphene. VI. an exact diagonalization study at nonzero integer filling. *Phys. Rev. B* **103**10.1103/physrevb.103.205416 (2021a).

[12] Kwan, Y. et al. Kekulé spiral order at all nonzero integer fillings in twisted bilayer graphene. *Phys. Rev. X* **11**10.1103/physrevx.11.041063 (2021).

[13] Christos M, Sachdev S, Scheurer MS (2022). Correlated insulators, semimetals, and superconductivity in twisted trilayer graphene. Phys. Rev. X.

[14] Xie, F., Regnault, N., Călugăru, D., Bernevig, B. A., and Lian, B. Twisted symmetric trilayer graphene. II. projected hartree-fock study. *Phys. Rev. B* **104**10.1103/physrevb.104.115167 (2021b).

[15] Ledwith, P. J. et al. TB or not TB? Contrasting properties of twisted bilayer graphene and the alternating twist *n*-layer structures (*n* = 3, 4, 5, … ). https://arxiv.org/abs/2111.11060 (2021).

[16] Wagner, G., Kwan, Y. H., Bultinck, N., Simon, S. H., and Parameswaran, S. Global phase diagram of the normal state of twisted bilayer graphene. *Phys. Rev. Lett*. **128**10.1103/physrevlett.128.156401 (2022). 10.1103/PhysRevLett.128.156401 35499897

[17] Wang, T. et al. Kekulé spiral order in magic-angle graphene: a density matrix renormalization group study. https://arxiv.org/abs/2211.02693 (2022).

[18] Kwan, Y. H. et al. Electron-phonon coupling and competing kekulé orders in twisted bilayer graphene. https://arxiv.org/abs/2303.13602 (2023).

[19] Wong D (2020). Cascade of electronic transitions in magic-angle twisted bilayer graphene. Nature.

[20] Zondiner U (2020). Cascade of phase transitions and dirac revivals in magic-angle graphene. Nature.

[21] Park JM, Cao Y, Watanabe K, Taniguchi T, Jarillo-Herrero P (2021). Tunable strongly coupled superconductivity in magic-angle twisted trilayer graphene. Nature.

[22] Hao Z (2021). Electric field–tunable superconductivity in alternating-twist magic-angle trilayer graphene. Science.

[23] Lin J-X (2022). Zero-field superconducting diode effect in small-twist-angle trilayer graphene. Nat. Phys..

[24] Morissette, E. et al. Dirac revivals drive a resonance response in twisted bilayer graphene. *Nat. Phys.*10.1038/s41567-023-02060-0 (2023).

[25] Cao Y, Park JM, Watanabe K, Taniguchi T, Jarillo-Herrero P (2021). Pauli-limit violation and re-entrant superconductivity in moirégraphene. Nature.

[26] Ojajärvi R, Hyart T, Silaev MA, Heikkilä TT (2018). Competition of electron-phonon mediated superconductivity and stoner magnetism on a flat band. Phys. Rev. B.

[27] Scheurer MS, Samajdar R (2020). Pairing in graphene-based moiré superlattices. Phys. Rev. Research.

[28] Lake, E., Patri, A. S., and Senthil, T. Pairing symmetry of twisted bilayer graphene: a phenomenological synthesis. *Phys. Rev. B* **106**10.1103/physrevb.106.104506 (2022).

[29] Christos M, Sachdev S, Scheurer MS (2020). Superconductivity, correlated insulators, and Wess-Zumino-Witten terms in twisted bilayer graphene. Proc. Natl Acad. Sci. USA.

[30] Khalaf, E., Chatterjee, S., Bultinck, N., Zaletel, M. P., & Vishwanath, A. Charged skyrmions and topological origin of superconductivity in magic-angle graphene. *Sci. Adv.* **7**10.1126/sciadv.abf5299 (2021). 10.1126/sciadv.abf5299 PMC8099185 33952523

[31] Khalaf E, Ledwith P, Vishwanath A (2022). Symmetry constraints on superconductivity in twisted bilayer graphene: Fractional vortices, 4*e* condensates, or nonunitary pairing. Phys. Rev. B.

[32] Scammell HD, Li JIA, Scheurer MS (2022). Theory of zero-field superconducting diode effect in twisted trilayer graphene. 2D Materials.

[33] Shavit G, Berg E, Stern A, Oreg Y (2021). Theory of correlated insulators and superconductivity in twisted bilayer graphene. Phys. Rev. Lett..

[34] Crépel V, Cea T, Fu L, Guinea F (2022). Unconventional superconductivity due to interband polarization. Phys. Rev. B.

[35] Wu F, MacDonald AH, Martin I (2018). Theory of phonon-mediated superconductivity in twisted bilayer graphene. Phys. Rev. Lett..

[36] Lian B, Wang Z, Bernevig BA (2019). Twisted bilayer graphene: a phonon-driven superconductor. Phys. Rev. Lett..

[37] Lewandowski C, Chowdhury D, Ruhman J (2021). Pairing in magic-angle twisted bilayer graphene: Role of phonon and plasmon umklapp. Phys. Rev. B.

[38] Lewandowski C, Nadj-Perge S, Chowdhury D (2021). Does filling-dependent band renormalization aid pairing in twisted bilayer graphene?. npj Quant. Mater..

[39] Yu, J., Xie, M., Wu, F., & Das Sarma, S. Euler obstructed cooper pairing in twisted bilayer graphene: nematic nodal superconductivity and bounded superfluid weight. 10.48550/arXiv.2202.02353 (2022).

[40] Phong VoT, Pantaleón PA, Cea T, Guinea F (2021). Band structure and superconductivity in twisted trilayer graphene. Phys. Rev. B.

[41] Wang, Y., Kang, J., and Fernandes, R. M. Topological and nematic superconductivity mediated by ferro-SU(4) fluctuations in twisted bilayer graphene. *Phys. Rev. B* **103**10.1103/physrevb.103.024506 (2021).

[42] Huang C, Wei N, Qin W, MacDonald AH (2022). Pseudospin paramagnons and the superconducting dome in magic angle twisted bilayer graphene. Phys. Rev. Lett..

[43] Fischer A, Klebl L, Honerkamp C, Kennes DM (2021). Spin-fluctuation-induced pairing in twisted bilayer graphene. Phys. Rev. B.

[44] Herzog-Arbeitman, J., Chew, A., Huhtinen, K.-E., Törmä, P., and Bernevig, B. A. Many-body superconductivity in topological flat bands. https://arxiv.org/abs/2209.00007 (2022).

[45] Cea, T. and Guinea, F. Coulomb interaction, phonons, and superconductivity in twisted bilayer graphene. *Proc. Natl Acad. Sci****.*** *USA.* **118**10.1073/pnas.2107874118 (2021). 10.1073/pnas.2107874118 PMC8364166 34362849

[46] Kozii, V., Zaletel, M. P., & Bultinck, N. Spin-triplet superconductivity from intervalley goldstone modes in magic-angle graphene. *Phys. Rev. B* **106**10.1103/physrevb.106.235157 (2022).

[47] You Y-Z, Vishwanath A (2019). Superconductivity from valley fluctuations and approximate so(4) symmetry in a weak coupling theory of twisted bilayer graphene. npj Quant. Mater..

[48] Oh M (2021). Evidence for unconventional superconductivity in twisted bilayer graphene. Nature.

[49] Kim H (2022). Evidence for unconventional superconductivity in twisted trilayer graphene. Nature.

[50] Poduval, P. P. and Scheurer, M. S. Vestigial singlet pairing in a fluctuating magnetic triplet superconductor: Applications to graphene moiré systems. https://arxiv.org/abs/2301.01344 (2023). 10.1038/s41467-024-45950-4 PMC10894192 38402211

[51] Brydon PMR, Das Sarma S, Hui H-Y, Sau JD (2014). Odd-parity superconductivity from phonon-mediated pairing: application to Cu_*x*_Bi_2_Se_3_. Phys. Rev. B.

[52] Scheurer MS (2016). Mechanism, time-reversal symmetry, and topology of superconductivity in noncentrosymmetric systems. Phys. Rev. B.

[53] Anderson PW, Brinkman WF (1973). Anisotropic superfluidity in ^3^He: a possible interpretation of its stability as a spin-fluctuation effect. Phys. Rev. Lett..

[54] Kozii V, Isobe H, Venderbos JWF, Fu L (2019). Nematic superconductivity stabilized by density wave fluctuations: possible application to twisted bilayer graphene. Phys. Rev. B.

[55] Gali V, Fernandes RM (2022). Role of electromagnetic gauge-field fluctuations in the selection between chiral and nematic superconductivity. Phys. Rev. B.

[56] Brydon, P. M. R., Agterberg, D. F., Menke, H., & Timm, C. Bogoliubov Fermi surfaces: general theory, magnetic order, and topology. *Phys. Rev. B* **98** https://arxiv.org/abs/1806.03773 (2018).

[57] Agterberg, D., Brydon, P., and Timm, C. Bogoliubov Fermi surfaces in superconductors with broken time-reversal symmetry. *Phys. Rev. Lett*. **118** https://arxiv.org/abs/1608.06461 (2017). 10.1103/PhysRevLett.118.127001 28388193

[58] Read N, Sachdev S (1990). Spin-Peierls, valence-bond solid, and Néel ground states of low-dimensional quantum antiferromagnets. Phys. Rev. B.

[59] Lee J, Sachdev S (2015). Wess-Zumino-Witten terms in graphene Landau levels. Phys. Rev. Lett..

[60] Liu X (2022). Visualizing broken symmetry and topological defects in a quantum Hall ferromagnet. Science.

[61] Chen, C. et al. Strong inter-valley electron-phonon coupling in magic-angle twisted bilayer graphene. https://arxiv.org/abs/2303.14903 (2023). 10.1038/s41586-024-08227-w PMC11634764 39663492

[62] Samajdar R (2021). Electric-field-tunable electronic nematic order in twisted double-bilayer graphene. 2D Mater..

[63] Zou L, Po HC, Vishwanath A, Senthil T (2018). Band structure of twisted bilayer graphene: Emergent symmetries, commensurate approximants, and wannier obstructions. Phys. Rev. B.

[64] Scheurer MS, Agterberg DF, Schmalian J (2017). Selection rules for cooper pairing in two-dimensional interfaces and sheets. npj Quant. Mater..

[65] Yip S, Garg A (1993). Superconducting states of reduced symmetry: General order parameters and physical implications. Phys. Rev. B.

[66] Bistritzer R, MacDonald AH (2011). Moiré bands in twisted double-layer graphene. Proc. Natl Acad. Sci. USA.

[67] Basko DM, Aleiner IL (2008). Interplay of coulomb and electron-phonon interactions in graphene. Phys. Rev. B.

[68] Basko, D. M. & Aleiner, I. L. Interplay of Coulomb and electron-phonon interactions in graphene. *Phys. Rev. B* **77**10.1103/physrevb.77.041409 (2008a).

[69] Putzer, B., Christos, M., & Scheurer, M. S. In preparation. (2023).

[70] Liu, C.-X., Chen, Y., Yazdani, A., & Bernevig, B. A. Electron-K-Phonon Interaction In Twisted Bilayer Graphene. https://arxiv.org/abs/2303.15551 (2023).

[71] Scheurer, M. S. *Mechanism, Symmetry and Topology of Ordered Phases in Correlated Systems*. Ph.D. Thesis, Karlsruher Institut für Technologie (KIT) (2016b).

[72] Timmons EI (2020). Electron irradiation effects on superconductivity in Pdte_2_: an application of a generalized anderson theorem. Phys. Rev. Res..

