# Supplementary material for: Nodal band-off-diagonal superconductivity in twisted graphene superlattices
Source: Nat Commun. 2023 Nov 6;14:7134. doi: 10.1038/s41467-023-42471-4 (PMC10628137; doi:10.1038/s41467-023-42471-4)
Supplement: Supplementary file 1 — Supplementary Information [file 41467_2023_42471_MOESM1_ESM.pdf]

Supplementary Information on Nodal band-off-diagonal superconductivity in twisted graphene superlattices

Maine Christos,[1] Subir Sachdev,[1] and Mathias S. Scheurer[2,3]

[1]Department of Physics, Harvard University, Cambridge MA 02138, USA
[2]Institute for Theoretical Physics, University of Innsbruck, Innsbruck A-6020, Austria
[3]Institute for Theoretical Physics III, University of Stuttgart, 70550 Stuttgart, Germany

SI A. NORMAL-STATE

1. Parallel and anti-parallel spins

We first discuss in more detail the spin structure of the superconducting states and the meaning of the symmetries of the effectively spinless bands, used in the main text to classify the superconducting states. We distinguish the two cases of (i) parallel spins in the two valleys and (ii) anti-parallel spins. To understand the physical meaning of the spinless symmetries of the main text, we start by listing the symmetries and their representations on the continuum-model operators $\psi_{\rho,\ell,\eta,s}(\boldsymbol{r})$ and band-operators $d_{\boldsymbol{k},\alpha,\eta,s}$ before normal-state polarization, where ρ, ℓ, η, s, and α are indices for the sublattice, layer, valley, spin, and the two flat bands, while ρ_j, η_j, s_j, and σ_j are Pauli matrices in sublattice, valley, spin, and band space, respectively:

1. Two-fold rotation along z, $C_{2z}: \psi(\boldsymbol{r}) \to \eta_x\rho_x\psi(-\boldsymbol{r})$ and $d_{\boldsymbol{k}} \to \eta_x d_{-\boldsymbol{k}}$
2. Spinless time-reversal, Θ: $\psi(\boldsymbol{r}) \to \eta_x\psi(\boldsymbol{r})$ and $d_{\boldsymbol{k}} \to \eta_x d_{-\boldsymbol{k}}$
3. Spinful time-reversal, Θ_s: $\psi(\boldsymbol{r}) \to \eta_x i s_y\psi(\boldsymbol{r})$ and $d_{\boldsymbol{k}} \to \eta_x i s_y d_{-\boldsymbol{k}}$
4. SO(3) spin-rotations, $R_s(\boldsymbol{\varphi})$: $\psi(\boldsymbol{r}) \to e^{i\boldsymbol{\varphi}\cdot\boldsymbol{s}/2}\psi(\boldsymbol{r})$ and $d_{\boldsymbol{k}} \to e^{i\boldsymbol{\varphi}\cdot\boldsymbol{s}/2}d_{\boldsymbol{k}}$
5. Global U(1) gauge symmetry, $U(\phi)$: $\psi(\boldsymbol{r}) \to e^{i\phi}\psi(\boldsymbol{r})$ and $d_{\boldsymbol{k}} \to e^{i\phi}d_{\boldsymbol{k}}$
6. Three-fold rotation along z, C_{3z}: $\psi(\boldsymbol{r}) \to e^{i\frac{2\pi}{3}\rho_z\eta_z}\psi(C_{3z}\boldsymbol{r})$ and $d_{\boldsymbol{k}} \to d_{C_{3z}\boldsymbol{k}}$
7. Two-fold rotation along x, C_{2x}: $\psi(\boldsymbol{r}) \to \rho_x\psi(C_{2x}\boldsymbol{r})$ and $d_{\boldsymbol{k}} \to \sigma_z d_{C_{2x}\boldsymbol{k}}$

Except for Θ_s and Θ, which are anti-linear, all representations are linear. In case (i) and assuming for concreteness that the active bands at the Fermi level of the flat bands are entirely spin-up ($s=\uparrow$), we simply define the fermionic operators of the main text as

$$c_{\boldsymbol{k},\alpha,\eta} := d_{\boldsymbol{k},\alpha,\eta,\uparrow}. \tag{S1}$$

The remaining (non-trivial) symmetries then act as $C_{2z} : c_{\boldsymbol{k}} \to \eta_x c_{-\boldsymbol{k}}$, $\Theta : c_{\boldsymbol{k}} \to \eta_x c_{-\boldsymbol{k}}$, $U(\phi) : c_{\boldsymbol{k}} \to e^{i\phi}c_{\boldsymbol{k}}$, $C_{3z} : c_{\boldsymbol{k}} \to c_{C_{3z}\boldsymbol{k}}$, and $C_{2x} : c_{\boldsymbol{k}} \to \sigma_z c_{C_{2x}\boldsymbol{k}}$, exactly as in the main text.

The situation is more non-trivial in case (ii). Let us assume, for notational simplicity, that the spin polarization of the active flat bands in valley $\eta=+$ is $s=\uparrow$ and in valley $\eta=-$ is $\downarrow$. Accordingly, we define

$$c_{\boldsymbol{k},\alpha,+} := d_{\boldsymbol{k},\alpha,+,\uparrow}, \quad c_{\boldsymbol{k},\alpha,-} := d_{\boldsymbol{k},\alpha,-,\downarrow}, \tag{S2}$$

as the effectively spinless fermionic operators used in the main text. It clearly holds, exactly as before, $U(\phi) : c_{\boldsymbol{k}} \to e^{i\phi}c_{\boldsymbol{k}}$, $C_{3z} : c_{\boldsymbol{k}} \to c_{C_{3z}\boldsymbol{k}}$, and $C_{2x} : c_{\boldsymbol{k}} \to \sigma_z c_{C_{2x}\boldsymbol{k}}$. However, Θ and C_{2z} are explicitly broken and, thus, have to be replaced by appropriate combinations with other symmetries. Let us define

$$\widetilde{\Theta} := U(-\pi/2)\Theta_s R_s(\pi\hat{\boldsymbol{e}}_z), \quad \widetilde{C}_{2z} := U(-\pi/2)C_{2z}R_s(\pi\hat{\boldsymbol{e}}_x), \tag{S3}$$

which are symmetries of the system and obey the same algebraic relations as the symmetries in the main text,

$$\widetilde{\Theta}^2 = \widetilde{C}_{2z}^2 = \mathbb{1}, \quad [\widetilde{\Theta}, \widetilde{C}_{2z}] = 0, \quad [\widetilde{\Theta}, C_{2x}] = [\widetilde{\Theta}, C_{3z}] = [\widetilde{C}_{2z}, C_{2x}] = [\widetilde{C}_{2z}, C_{3z}] = 0. \tag{S4}$$

In fact, their representation on the fermions defined in Eq. (S2) is exactly the same as that of Θ and C_{2z} in the main text, $\widetilde{C}_{2z}: c_{\boldsymbol{k}} \to \eta_x c_{-\boldsymbol{k}}$ and $\widetilde{\Theta}: c_{\boldsymbol{k}} \to \eta_x c_{-\boldsymbol{k}}$. As such, for case (ii), the time-reversal symmetry Θ and two-fold-rotational symmetry C_{2z} in the main text can be identified with $\widetilde{\Theta}$ and $\widetilde{C}_{2z}$ in Eq. (S3). To illustrate this further and also explicitly discuss the spin structure of the order parameter, we transform the superconducting order parameter back to the d-fermions via Eq. (S2),

$$\mathcal{H}_{\rm p} = \sum_{\boldsymbol{k}} c^\dagger_{\boldsymbol{k},\alpha,+}(\Delta_{\boldsymbol{k}})_{\alpha,\alpha'} c^\dagger_{-\boldsymbol{k},\alpha',-} = \frac{1}{2}\sum_{\boldsymbol{k}} d^\dagger_{\boldsymbol{k},\alpha,+,s}[(s_0+s_z)is_y]_{s,s'}(\Delta_{\boldsymbol{k}})_{\alpha,\alpha'} d^\dagger_{-\boldsymbol{k},\alpha',-,s'}, \tag{S5}$$

which shows that we obtain an admixture of singlet and (unitary) triplet pairing. To demonstrate the action of $\widetilde{\Theta}$ and $\widetilde{C}_{2z}$ more explicitly and provide a consistency check, let us focus on $\Delta_{\boldsymbol{k}} = 2\Delta\sigma_y$, where Eq. (S5) becomes

$$\mathcal{H}_{\rm p} = \frac{\Delta}{2}\sum_{\boldsymbol{k}} d^\dagger_{\boldsymbol{k}}(is_0\eta_y + s_z\eta_x)is_y\sigma_y d^\dagger_{-\boldsymbol{k}}. \tag{S6}$$

From Eq. (S3), we find the representations $\widetilde{C}_{2z}: d_{\boldsymbol{k}} \to \eta_x s_x d_{-\boldsymbol{k}}$ and $\widetilde{\Theta}: d_{\boldsymbol{k}} \to \eta_x s_x d_{-\boldsymbol{k}}$; applying this in Eq. (S6), we find that

$$\widetilde{C}_{2z}: \Delta \to \Delta, \quad \widetilde{\Theta}: \Delta \to -\Delta^*, \tag{S7}$$

exactly as in the main text.

For case (i), Eq. (S5) instead becomes

$$\mathcal{H}_{\rm p} = \sum_{\boldsymbol{k}} c^\dagger_{\boldsymbol{k},\alpha,+}(\Delta_{\boldsymbol{k}})_{\alpha,\alpha'} c^\dagger_{-\boldsymbol{k},\alpha',-} = \frac{1}{2}\sum_{\boldsymbol{k}} d^\dagger_{\boldsymbol{k},\alpha,+,s}[(s_x+is_y)is_y]_{s,s'}(\Delta_{\boldsymbol{k}})_{\alpha,\alpha'} d^\dagger_{-\boldsymbol{k},\alpha',-,s'}, \tag{S8}$$

i.e., a non-unitary triplet state—as expected [1] since this is the "Hund's partner" of the singlet-triplet admixed state in Eq. (S5), obtained by an independent spin-rotation in the two valleys [SU(2)$_-\times$ SU(2)$_+$]. For $\Delta_{\boldsymbol{k}} = 2\Delta\sigma_y$ this yields

$$\mathcal{H}_{\rm p} = \frac{\Delta}{2}\sum_{\boldsymbol{k}} d^\dagger_{\boldsymbol{k}}(s_x\eta_x + is_y\eta_x)is_y\sigma_y d^\dagger_{-\boldsymbol{k}}. \tag{S9}$$

Again in accordance with the spinless formulation of the main text, we get $C_{2z}: \Delta \to \Delta$ and $\Theta: \Delta \to -\Delta^*$.

We finally note that the normal-state polarization also determines the spin-structure of the fluctuating orders in Table II and Table S1: switching between the two scenarios (i) and (ii) requires replacing an order parameter for the correlated insulator by its "Hund's partner" (see, e.g., Table II in [2] for a complete list). As the system is believed to be close to the SU(2)$_-\times$ SU(2)$_+$ symmetric limit (the intervalley Hund's coupling was estimated to be smaller than 0.1 meV in [3]), the strength of fluctuations of Hund's partners is expected to be roughly the same. As such, both scenarios (i) and (ii) are consistent with a mechanism based on fluctuations of an order parameter of a correlated insulator. As mentioned in the main text, this is different for phonons, where only scenario (i) allows for intervalley phonons providing the pairing glue.

2. Hartree-Fock numerics

To capture the non-interacting band structure, we use a continuum-model description [4],

$$\mathcal{H}_0 = \int \mathrm{d}\boldsymbol{r}\, \psi^\dagger_{\rho,\ell,\eta,s}(\boldsymbol{r})\,[h_\eta(\boldsymbol{\nabla},\boldsymbol{r})]_{\rho,\ell;\rho',\ell'}\,\psi_{\rho',\ell',\eta,s}(\boldsymbol{r}), \tag{S10}$$

where $\psi^\dagger_{\rho,\ell,\eta,s}$ creates an electron of spin $s =\uparrow,\downarrow$, in valley $\eta = \pm$, sublattice $\rho = A, B$, and with pseudo-layer quantum-number $\ell = 1, 2$; in case of TBG, ℓ refers to the actual two graphene layers, whereas, for TTG, it denotes the two mirror-even layer-eigenstates, $(1,1,1)^T$ and $(1,-2,1)^T$, of the three layers [5]. The continuum model involves two terms, $(h_\eta)_{\ell,\ell'} = \delta_{\ell,\ell'}h^{(d)}_{\ell,\eta}(\boldsymbol{\nabla}) + (h^{(t)}_\eta(\boldsymbol{r}))_{\ell,\ell'}$; the first one, $h^{(d)}_{\ell,\eta} = -i\hbar v_F e^{i\frac{\rho_z\theta_\ell}{2}}(\eta\rho_x\partial_x - \rho_y\partial_y)e^{-i\frac{\rho_z\theta_\ell}{2}}$ with ρ_j being Pauli matrices in sublattice space, describes the Dirac cones of chirality η, rotated by $\theta_\ell = (-1)^\ell\theta/2$ in the two (pseudo)layers ℓ; the second one, $h^{(t)}$, captures the tunneling between the layers, with amplitude w_0 and w_1

between the same and opposite sublattices, respectively. The modulation of the tunneling on the moiré scale leads to a reconstruction of the band structure, exhibiting nearly flat bands for magic angles around $\theta \simeq 1.1^\circ$ and $\theta \simeq 1.5^\circ$ for TBG and TTG, respectively. We take $w_1 = 89$ meV, $\frac{w_0}{w_1} = .55$, $v_F = 10^6$ m/s, $\theta = 1.09^\circ$ in all our numerical calculations.

As already mentioned above, experiments [6, 7] indicate that the superconducting phase in the density regime $2 < |\nu| < 3$ coexists with the reset behavior at half-filling, $|\nu| = 2$, of the upper or low flat-bands. To model this effect, we add Coulomb repulsion,

$$\mathcal{H}_C = \frac{1}{2N} \sum_{\boldsymbol{q}} V(\boldsymbol{q}) \rho_{\boldsymbol{q}} \rho_{-\boldsymbol{q}} \tag{S11}$$

to our Hamiltonian, where $\rho_{\boldsymbol{q}}$ is the Fourier transform of the density of the continuum-model electrons $c_{\boldsymbol{r}}$ and the N the number of moiré unit cells. We assume a double gate screened Coulomb potential of the form:

$$V(\boldsymbol{q}) = \frac{1}{A_m} \frac{1 - e^{-2d_s|\boldsymbol{q}|}}{2\epsilon\epsilon_0 |\boldsymbol{q}|} \tag{S12}$$

In the above, A_m is the area of a real-space moiré unit cell (since we consider TBG and not TTG in our numerics, we take A_m to be the moiré unit cell for 1.09°), d_s is the screening distance which we take to be 40 nm, and ϵ is the dielectric constant we take to be $\epsilon \simeq 4$. Note that projecting Eq. (S11) into the bands of TTG will also lead to interactions coupling the mirror-sectors. However, as was shown [2] analytically in a specific limit and numerically for realistic parameters, also the interacting physics of TTG decays into that of the TBG and that of a single Dirac cone for $D_0 = 0$. As such, it is justified to focus on the mirror-bands as in Eq. (S10) when discussing the reset physics in TTG at $D_0 = 0$.

In computing the normal state, we assume the same normal state density matrix as in Ref. 2 where the expectation value $\langle c^\dagger_{\boldsymbol{k},\alpha,\eta} c_{\boldsymbol{k},\beta,\eta} \rangle$ is equal to the $\frac{1}{2}$Id in the subspace of the flat bands of one spin flavor which are half filled in our normal state and equal to Id in the flat bands of the remaining spin flavor which are fully polarized. We emphasize that we are assuming a static, momentum independent ansatz for the normal state density matrix which is not obtained self consistently. As can be seen in Fig. II, instead of just rigidly shifting one spin species away from the Fermi level, there are also significant band renormalizations, in particular for the active spin flavor. Similar to the toy model with $t' < 0$ used in Fig. 1, the Dirac cones at the K and K' points are pushed towards the top of the bands.

3. Gauge Fixing

We will also describe how we fix the phases of the continuum model Bloch wavefunctions we use in our computations. We denote the wavefunction of band n in valley η at momentum $\boldsymbol{k}$ by $u_{\boldsymbol{k},n,\eta}$. We use $C_{2z}\mathcal{T}$ to fix the phase of the wavefunctions to be either $+1$ or -1 by enforcing:

$$C_{2z}\mathcal{T} u_{\boldsymbol{k},n,\eta} = u_{\boldsymbol{k},n,\eta} \tag{S13}$$

We then fix the relative sign of wavefunctions in opposite flat bands but the same valley with the chiral symmetry operator C as:

$$C u_{\boldsymbol{k},\pm,\eta} = i\eta \pm u_{\boldsymbol{k},\mp,\eta} / |\langle u^*_{\boldsymbol{k},\mp,\eta}| C |u_{\boldsymbol{k},\pm,\eta}\rangle| \tag{S14}$$

We fix the relative sign of wavefunctions in opposite bands and opposite valleys with PHC_{2z}, where PH a unitary particle hole symmetry operator with:

$$PHC_{2z} u_{\boldsymbol{k},\pm,\eta} = \pm\eta u_{\boldsymbol{k},\mp,-\eta} \tag{S15}$$

Finally, we use time-reversal symmetry to fix the relative sign between wavefunctions at opposite $\boldsymbol{k}$, in opposite valleys, but within the same band:

$$\mathcal{T} u_{\boldsymbol{k},n,\eta} = u_{-\boldsymbol{k},n,-\eta} \tag{S16}$$

SI B. GAP EQUATION AT $T = 0$

In this appendix we will discuss the self consistency equations we solve to obtain our $T = 0$ solutions. In general, we write the Hamiltonian in a Nambu basis as:

$$\mathcal{H}_{\boldsymbol{k}} = \begin{pmatrix} c^\dagger_{\boldsymbol{k},+} & c_{-\boldsymbol{k},-} \end{pmatrix} \begin{pmatrix} \xi_{\boldsymbol{k},+} & \Delta(\boldsymbol{k}) \\ \Delta(\boldsymbol{k})^\dagger & -\xi_{-\boldsymbol{k},-} \end{pmatrix} \begin{pmatrix} c_{\boldsymbol{k},+} \\ c^\dagger_{-\boldsymbol{k},-} \end{pmatrix} \tag{S17}$$

Where we have suppressed spin and band indices, and both $\xi_{\boldsymbol{k},\pm}$ and $\Delta_{\boldsymbol{k}}$ are matrices in band and spin space. $\xi_{\boldsymbol{k},\pm}$ represents the normal state dispersion in the $\pm$ valleys, which we take to be spin polarized and renormalized by Coulomb interactions as described in App. SI A. $\Delta_{\boldsymbol{k}}$ can be expressed as:

$$\Delta^{\alpha,\eta;\beta,-\eta}_{\boldsymbol{k}} = \frac{1}{N}\sum_{\boldsymbol{k},\boldsymbol{k'}} \chi_{\boldsymbol{k},\boldsymbol{k'}} \lambda^{\alpha,\eta;\gamma,\eta'}_{\boldsymbol{k},\boldsymbol{k'}} \left(\langle c_{-\boldsymbol{k}} c_{\boldsymbol{k}}\rangle^T\right)^{\gamma,\eta';\delta,-\eta'} \left(\lambda^T_{-\boldsymbol{k},\boldsymbol{k'}}\right)^{\delta,-\eta';\beta,-\eta} \tag{S18}$$

In the above, $\lambda^{\alpha,\eta;\gamma,\eta'}_{\boldsymbol{k},\boldsymbol{k'}}$ represent form factors of some matrix elements which could represent either phonons or fluctuations projected into the flat bands and may be valley diagonal or off diagonal. $V_{\boldsymbol{k},\boldsymbol{k'}}$ is an isotropic potential which we will generally take to be attractive and flat for phonons and attractive with some lorentzian form for fluctuation mediated pairing. Since we will be assuming interactions with strength less than the scale of the coulomb interactions, we will treat the polarized spin flavor which is fully occupied at $\nu = 2$ as a spectator and assume the pairing is zero in these bands. The self consistency condition we solve at $T = 0$ is:

$$\langle c_{-\boldsymbol{k},\alpha,-} c_{\boldsymbol{k},\beta,+}\rangle = U^*_{\boldsymbol{k}} \chi_{\boldsymbol{k}} U^T_{\boldsymbol{k}} \tag{S19}$$

Where $U_{\boldsymbol{k}}$ is defined as the unitary operator such that:

$$U^\dagger_{\boldsymbol{k}} \mathcal{H}_{\boldsymbol{k}} U_{\boldsymbol{k}} = D_{\boldsymbol{k}} \tag{S20}$$

Here, $D_{\boldsymbol{k}}$ is a diagonal matrix with the Fermi-Dirac functions of eigenvalues of $\mathcal{H}_{\boldsymbol{k}}$ at $T = 0$ as its diagonal entries. $\chi_{\boldsymbol{k}}$ is the matrix with Fermi-Dirac functions at $T = 0$ K of the entries of D on the diagonal. We also must impose Fermi-Dirac statistics as a constraint on our solutions. We enforce this constraint at each iteration by splitting $\langle c_{-\boldsymbol{k},\alpha,-} c_{\boldsymbol{k},\beta,+}\rangle$ into components which go as either η_x in valley space (denoted $E_{\boldsymbol{k}}$) or η_y in valley space (denoted as $O_{\boldsymbol{k}}$) depending on whether the pairing is even or odd under $\boldsymbol{k} \to -\boldsymbol{k}$ and the antisymmetry or symmetry of the band indices as:

$$O_{\boldsymbol{k}} = \frac{1}{2}\left(\langle c_{-\boldsymbol{k},\alpha,-} c_{\boldsymbol{k},\beta,+}\rangle + \langle c_{\boldsymbol{k},\beta,-} c_{-\boldsymbol{k},\alpha,+}\rangle\right) \qquad E_{\boldsymbol{k}} = \frac{1}{2}\left(\langle c_{-\boldsymbol{k},\alpha,-} c_{\boldsymbol{k},\beta,+}\rangle - \langle c_{\boldsymbol{k},\beta,-} c_{-\boldsymbol{k},\alpha,+}\rangle\right) \tag{S21}$$

Our iterative procedure then proceeds as follows. At the zeroth iteration, an ansatz for $\langle c_{-\boldsymbol{k},\alpha,-} c_{\boldsymbol{k},\beta,+}\rangle$ satisfying the desired symmetries is selected. Then at each iteration, the chemical potential is adjusted to give the desired filling, which we take to be $\nu = 2.5$ in our numerics. $U_{\boldsymbol{k}}$ and the resulting functions $O_{\boldsymbol{k}}$ and $E_{\boldsymbol{k}}$ are then computed and plugged back into $\Delta_{\boldsymbol{k}}$, (which also is guaranteed to obey Fermi-Dirac statistics assuming our generalized form factors obey time reversal symmetry). $\Delta_{\boldsymbol{k}}$ is then used to compute the new $U_{\boldsymbol{k}}$, and the procedure is repeated until convergence is reached in $\Delta_{\boldsymbol{k}}$ and μ. In practice, in our $T = 0$ numerics, we take $\boldsymbol{q} = \boldsymbol{k} - \boldsymbol{k'}$ to only be summed over the first Brillouin zone when we consider fluctuation mediated superconductivity, an assumption justified for our fluctuation mediated SC by $\chi(\boldsymbol{q})$ falling off as $\frac{1}{|\boldsymbol{q}|^2}$ near the first Brillouin zone edge. For phonon mediated superconductivity, we include an additional shell of the 6 nearest Brillouin zones in our sum over $\boldsymbol{q}$. Including more shells may reduce the needed coupling, though we expect the leading instability of A_1 phonons should be unchanged.

SI C. LINEARIZED GAP EQUATION AT T_c

In this appendix, we will describe how we compute solutions to the linearized gap equation at T_c. As in App. SI B, we will assume a spin polarized normal state and only consider superconducting instabilities within a single spin flavor. We recall that for the case of fluctuation-mediated superconductivity, we couple electrons to bosonic modes ($j = 1, 2, \dots$) as, e.g., in Eq. (4), with $\lambda^j_{\alpha,\eta;\alpha',\eta'}$ capturing the symmetries broken by the corresponding order parameter. In order to compactly write down the linearized gap equation, it is convenient to express λ^j as

$$\left(\lambda^j_{\alpha,\eta;\alpha',\eta'}\right)_{\boldsymbol{k},\boldsymbol{k'}} = A^{\alpha,\eta;\alpha',-\eta}_{\boldsymbol{k},\boldsymbol{k'}} \delta_{\eta,-\eta'} + B^{\alpha,\eta;\alpha',\eta}_{\boldsymbol{k},\boldsymbol{k'}} \delta_{\eta,\eta'}. \tag{S22}$$

TABLE S1: Generalization of Table II of the main text, where we also indicate the dominant superconducting orders ($\bar{\Delta}$) in the microscopic basis, obtained by applying Eq. (7) in the sublattice basis. The phonon modes refer to the sublattice-basis form $\bar{\lambda}_j$ of the coupling, cf. Eq. (8), and "g-nematic" stands for the (intravalley) graphene nematic state of Ref. 8, which has the same coupling as the E_2 phonon.

Fluctuating Order			Leading SC (band)		Leading SC (microscopic)	
type	λ^j	$\bar{\lambda}^j$	$\Delta_{\boldsymbol{k},\eta}$	IR	$\bar{\Delta}_{\boldsymbol{k},\eta}$	IR
T-IVC/A_1, B_1 phonon	$\sigma_0\eta_{x,y}$	$\rho_x\eta_{x,y}$	$\sigma_y\delta_{\boldsymbol{k}}$	A_2	$\rho_z\eta\delta_{\boldsymbol{k}}$	A_2
K-IVC	$\sigma_y\eta_{x,y}$	$\rho_y\eta_{x,y}$	$\sigma_0\eta\delta_{\boldsymbol{k}}$	B_1	$\rho_0\eta\delta_{\boldsymbol{k}}$	B_1
SLP+	$\sigma_y\eta_z$	$\rho_z\eta_0$	$\sigma_y\delta_{\boldsymbol{k}},\ \sigma_0\eta\delta_{\boldsymbol{k}}$	$A_2,\ B_1$	$\rho_z\eta\delta_{\boldsymbol{k}},\ \rho_0\eta\delta_{\boldsymbol{k}}$	$A_2,\ B_1$
SLP−	$\sigma_y\eta_0$	$\rho_z\eta_z$	$\sigma_x\eta\delta_{\boldsymbol{k}},\ \sigma_z\eta\delta_{\boldsymbol{k}}$	$B_2,\ B_1$	$(\rho_y, \rho_x\eta)$	E_1
N-IVC	—	$\eta_{x,y}\rho_{0,z}$	—	—	$(\rho_y, \rho_x\eta)$	E_1
g-nematic/E_2 phonon	—	$(\eta_z\rho_y, -\rho_x)$	—	—	$\rho_0\eta\delta_{\boldsymbol{k}}$	B_1

Here we also include the momentum dependence of the matrix elements, which arises when we study phonons and order parameter fluctuations projected from the sublattice basis to the band basis. In Eq. (S22), $A_{\boldsymbol{k},\boldsymbol{k}'}$ are the valley off diagonal pieces of the form factor $\lambda^j_{\alpha,\eta;\alpha',\eta'}$ and $B_{\boldsymbol{k},\boldsymbol{k}'}$ is the valley diagonal pieces. With this notation in hand, the linearized gap equation we solve is

$$\left(\Delta(\boldsymbol{k})^\dagger\right)^{\alpha,-;\beta+} = \sum_{\boldsymbol{q}} \chi_{\boldsymbol{q}} \left(\mathcal{G}^{\delta-;\gamma+}_{\boldsymbol{k}-\boldsymbol{q}} B^{\gamma+;\beta+}_{\boldsymbol{k}-\boldsymbol{q},\boldsymbol{q}} (B^T_{-\boldsymbol{k}+\boldsymbol{q},-\boldsymbol{q}})^{\alpha-;\delta-} - \mathcal{G}^{\gamma-;\delta+}_{-\boldsymbol{k}+\boldsymbol{q}} A^{\gamma-;\beta+}_{\boldsymbol{k}-\boldsymbol{q},\boldsymbol{q}} (A^T_{-\boldsymbol{k}+\boldsymbol{q},-\boldsymbol{q}})^{\alpha-;\delta+} \right), \tag{S23}$$

where the Greens function $\mathcal{G}^{\alpha+;\beta-}_{\boldsymbol{k}-\boldsymbol{q}}$ defined by

$$\begin{aligned}\mathcal{G}^{\alpha+;\beta-}_{\boldsymbol{k}} = \frac{1}{2A_m} \Bigg(& \frac{\Delta^{00}_{\boldsymbol{k}}}{2|\xi_{\boldsymbol{k},0}|} \left(1 - 2n_F(|\xi_{\boldsymbol{k},0}|)\right) (\sigma_0 + \sigma_z)^{\alpha\beta} + \frac{\Delta^{11}_{\boldsymbol{k}}}{2|\xi_{\boldsymbol{k},1}|} \left(1 - 2n_F(|\xi_{\boldsymbol{k},1}|)\right) (\sigma_0 - \sigma_z)^{\alpha\beta} + \\ & \frac{\Delta^{01}_{\boldsymbol{k}}}{\xi_{\boldsymbol{k},0} + \xi_{\boldsymbol{k},1}} (n_F(-\xi_{\boldsymbol{k},1}) - n_F(\xi_{\boldsymbol{k},0}))(\sigma_x + i\sigma_y)^{\alpha\beta} + \frac{\Delta^{10}_{\boldsymbol{k}}}{\xi_{\boldsymbol{k},0} + \xi_{\boldsymbol{k},1}} (n_F(-\xi_{\boldsymbol{k},1}) - n_F(\xi_{\boldsymbol{k},0}))(\sigma_x - i\sigma_y)^{\alpha\beta} \Bigg).\end{aligned} \tag{S24}$$

Here $\Delta^{\alpha\beta}_{\boldsymbol{k}}$ denote the pairing in band space where $\alpha, \beta = 0, 1$ label the upper and lower flat band. Finding a solution to the above equation then amounts to computing the right-hand side of Eq. (S23), diagonalizing it in the space of momenta, Nambu index, and band index, and looking at the eigenvectors which attain eigenvalue 1 for some value of T. To enforce Fermi-Dirac statistics, we solve the above equation on half of the moiré Brillouin zone. We also exclude the edge points in our linearized gap equation computations for phonons and projected order fluctuations. We expect including these points would reduce the needed coupling to obtain a finite T_c (or reduce T_c for fixed coupling) but not change the leading instabilities.

SI D. ADDITIONAL STATEMENTS ABOUT SUPERCONDUCTIVITY AND PHONONS

1. Generalization to sublattice basis

Due to the basis independent form of the (anti)commutator relation in Eq. (7), it can be readily applied in any basis. As we also study in the numerics of the main text momentum-independent coupling matrices $\bar{\lambda}_j$ in the microscopic sublattice basis, it seems natural to also apply the commutator relation in that basis. Upon noting that the additional projection onto the flat bands does, in general, not commute with the order parameters, it is clear that applying Eq. (7) can only provide approximate guidance even in the strict flat-band limit. Notwithstanding these approximations, the results, summarized in Table S1, agree well with the numerics shown in Fig. 4 of the main text. In the case of N-IVC fluctuations, the listed E_1 superconductor is the option where the highest number of components obey Eq. (7), while all components obey it in all other cases.

2. Electron phonon coupling in TTG

As it exhibits three layers, the discussion of the layer structure of the phonon modes in TTG requires additional comments. Starting from uncoupled optical A_1, B_1, and E_2 phonons in the three layers of TTG, we can decompose each of these modes into two mirror-even ($\mu = e_1, e_2$) and one mirror-odd ($\mu = o$) contributions,

$$\boldsymbol{v}_{e_1} = \frac{1}{\sqrt{3}}\begin{pmatrix}1\\1\\1\end{pmatrix}, \quad \boldsymbol{v}_{e_2} = \frac{1}{\sqrt{6}}\begin{pmatrix}1\\-2\\1\end{pmatrix}, \quad \boldsymbol{v}_0 = \frac{1}{\sqrt{2}}\begin{pmatrix}1\\0\\-1\end{pmatrix}. \tag{S25}$$

Upon projection into the mirror-even electronic sectors, forming the relevant low-energy flat-band degrees of freedom, the mode $\boldsymbol{v}_0$ vanishes completely (due to mirror-symmetry), while the first two survive. Their respective projected coupling is of the form of Eq. (8) with $\boldsymbol{v}_{e_1} = (1,1)^T/\sqrt{3}$ and $\boldsymbol{v}_{e_2} = (1,-2)^T/\sqrt{6}$.

3. Electron-phonon matrix elements

In this appendix, we analyze the momentum-independent terms of the electron-phonon coupling matrices $\lambda^{g,j,\mu}_{\boldsymbol{k},\alpha,\eta;\boldsymbol{k}',\alpha'\eta'}$ in Eq. (9). As a result of C_{3z} symmetry, the coupling terms of E_2 cannot have a momentum-independent component and so we focus on $g = A_1, B_1$. Let us expand in Pauli matrices in band and valley space,

$$\lambda^{g,\mu}_{\boldsymbol{k},\alpha,\eta;\boldsymbol{k}',\alpha'\eta'} = \sum_{j_1,j_2} c^{g,\mu}_{j_1,j_2}(\sigma_{j_1})_{\alpha,\alpha'}(\eta_{j_2})_{\eta,\eta'} + \mathcal{O}(\boldsymbol{k},\boldsymbol{k}'), \tag{S26}$$

where Hermiticity implies $c^{g,\mu}_{j_1,j_2} \in \mathbb{R}$. The combination of $U(1)_v$ (valley-charge conservation), C_{2z}, and Θ implies that only $c^{A_1,\mu}_{j_1,x}$, $c^{B_1,\mu}_{j_1,y}$, $j_1 = 0,x,z$ can be non-zero. Chiral symmetry C has the representation ρ_z and $\eta_z\sigma_y$ in the sublattice and band basis, respectively. As ρ_z anti-commutes with both $\Lambda_{A_1} = \eta_x\rho_x$ and $\Lambda_{B_1} = \eta_y\rho_x$, their band projections in Eq. (S26) also have to anti-commute with $\eta_z\sigma_y$; this leaves us with $c^{A_1,\mu}_{0,x}$ and $c^{B_1,\mu}_{0,y}$ as the only non-zero terms. Furthermore, the unitary particle-hole symmetry P anti-commutes with the layer-even ($\mu = +$) and commutes with the layer-odd ($\mu = -$) modes. Being represented by $-i\eta_z\sigma_y$, this is inconsistent with $c^{A_1,-}_{j_1,x}, c^{B_1,-}_{j_1,y} \neq 0$, which thus have to vanish. This is in line with our numerics, where we find very small projections of the layer-odd A_1 and B_1 modes. Their layer-even counterparts, however, are consistent with P if only $c^{A_1,\mu}_{0,x}$ and $c^{B_1,\mu}_{0,y}$ are non-zero. Taken together, we find

$$\lambda^{g,-}_{\boldsymbol{k},\alpha,\eta;\boldsymbol{k}',\alpha'\eta'} = \mathcal{O}(\boldsymbol{k},\boldsymbol{k}'),\ g = A_1, B_1, \quad \lambda^{A_1,+}_{\boldsymbol{k},\alpha,\eta;\boldsymbol{k}',\alpha'\eta'} = \sigma_0\eta_x + \mathcal{O}(\boldsymbol{k},\boldsymbol{k}'), \quad \lambda^{B_1,+}_{\boldsymbol{k},\alpha,\eta;\boldsymbol{k}',\alpha'\eta'} = \sigma_0\eta_y + \mathcal{O}(\boldsymbol{k},\boldsymbol{k}'). \tag{S27}$$

SI E. PAIRING FOR OTHER NORMAL-STATE ORDERS

In the main text, we have discussed pairing in the case of a spin polarized or spin-valley locked normal state. We here comment on the consequences for superconductivity for two other, plausible normal-state scenarios.

1. T-IVC & SP order

Given the current insights from experiment, the most natural alternative scenario is that the normal state exhibits both T-IVC [9] and spin polarization [3, 10] simultaneously. The projection to the remaining two active flavor degrees of freedom is given by

$$P_{\nu=2} = \frac{1}{4}(1+s_z)(1+\eta_x\rho_x). \tag{S28}$$

Increasing ν beyond $\nu = 2$ will lead to a metallic state with two non-degenerate bands $\alpha = \pm$ coming from the original flat-band manifold. Let us denote the associated creation operators by $c^\dagger_{\boldsymbol{k},\alpha}$, which have one index less than the associated operators discussed in the main text since valley is not a good quantum number anymore. The superconducting order parameter is a 2×2 matrix, coupling to the electrons as $\sum_{\boldsymbol{k},\alpha,\alpha'} c^\dagger_{\boldsymbol{k},\alpha}(\Delta_{\boldsymbol{k}})_{\alpha,\alpha'} c^\dagger_{-\boldsymbol{k},\alpha'} + \text{H.c.}$,

and thus has to obey $\Delta_{\boldsymbol{k}} = -\Delta^T_{-\boldsymbol{k}}$. As the projector in Eq. (S28) commutes with C_{2z} (in fact, also with C_{2x} and C_{3z}), all pairing states must still be either even or odd under C_{2z} (transform under one of the IRs of D_6 or C_6). Since Eq. (S28) projects onto the subspace where $\eta_x\rho_x$ is -1, it holds C_{2z}: $c^\dagger_{\boldsymbol{k},\alpha} \to -c^\dagger_{-\boldsymbol{k},\alpha}$ and, hence,

$$C_{2z}: \quad \Delta_{\boldsymbol{k}} \quad \longrightarrow \quad \Delta_{-\boldsymbol{k}} = -\Delta^T_{\boldsymbol{k}}, \tag{S29}$$

which is the analogue of Eq. (2) of the main text. As before, all C_{2z}-even states must be entirely band-off-diagonal, $\Delta_{\boldsymbol{k}} = \delta_{\boldsymbol{k}}\sigma_y$. However, since the number of active degrees of freedom is reduced, there are more restrictions: all C_{2z}-odd superconductors must have zeros in the Brillouin zone due to $\Delta_{\boldsymbol{k}} = -\Delta^T_{-\boldsymbol{k}} = -\Delta_{-\boldsymbol{k}}$.

For completeness and to conveniently address energetics, we extend the discussion to the microscopic sublattice basis. Let $\bar{\Delta}_{\boldsymbol{k}}$ be the corresponding superconducting order parameter—an 8×8 matrix in sublattice, valley, and spin space. Then pairings are constrained to obey

$$P_{\nu=2}\bar{\Delta}_{\boldsymbol{k}} s_y\eta_x P^T_{\nu=2} = \bar{\Delta}_{\boldsymbol{k}} s_y\eta_x. \tag{S30}$$

The order parameters which are compatible with Eq. (S30) will all be spin triplets. The C_{2z}-even states, i.e., order parameters transforming under A_2, E_2, or A_1, will have the form (suppressing $\boldsymbol{k}$-dependencies) $\bar{\Delta}_{\boldsymbol{k}} \sim P_{\nu=2}s_x\eta_z\rho_z$; in line with our symmetry arguments above, one can check that they will go as σ_y in band space and thus be purely band off diagonal in the subspace defined by $P_{\nu=2}$. The pairings which are odd under C_{2z} include the B_1 and B_2 pairings with $\bar{\Delta}_{\boldsymbol{k}} \sim P_{\nu=2}s_x\rho_0$, and E_1 pairings with $\bar{\Delta}_{\boldsymbol{k}} \sim P_{\nu=2}s_x(\rho_x, \rho_y\eta_z)$ previously discussed in our main text; however, as pointed out above and unlike in the main text, the C_{2z}-odd pairings in both the band basis and sublattice basis are no longer allowed to have a component without a sign change since only the momentum odd components of the B_1, B_2, and E_2 pairings survive projection $P_{\nu=2}$.

Since only the band-off-diagonal A_2 state can have a non-sign-changing order parameter, a superconducting state satisfying the criterion around Eq. (7) of the main text can only be this state (or none). We have studied which of the pairing mechanisms survive the projection and whether they favor or disfavor A_2 pairing, see Table S2. We find that A_1 phonons, T-IVC fluctuations, and spin fluctuations all provide an attractive pairing potential, and if any of these have large enough couplings to overcome the normal state band splitting, the A_2 triplet pairing is the leading instability, as in the main text. Furthermore, due to the fact that the remaining bands after reconstruction, as described by the projector $P_{\nu=2}$, are not degenerate (there is no remaining spin symmetry to guarantee degeneracy), a Bogoliubov Fermi surface or a fully gapped state and, thus, a transition from nodal to gapped as a function of filling are possible depending on parameters (similar to our discussion in the main text).

$\Delta_{\boldsymbol{k}}$	IR of D_6	T-IVC/A_1 phonon $\eta_x\rho_x$	quantum spin Hall $s_z\eta_z\rho_z$	spin polarized s_z	N-IVC $\eta_x(\rho_0, \eta_z\rho_z)$	quantum Hall $\eta_z\rho_z$
$P_{\nu=2}\,(s_x\eta_z\rho_z\delta_{\boldsymbol{k}})$	A_2	✓	✗	✓	✗	✗

TABLE S2: We list the possible pairing glues which are compatible with a T-IVC+SP normal state (i.e., the interactions survive projection to the space of the upper T-IVC bands of a single spin flavor). We denote interactions which will generate an attractive interaction for the A_2 pairing with a ✓ and interactions which will generate a repulsive interaction with a ✗.

2. T-IVC normal state

We will now consider a simpler normal state which leaves twice the number of degrees of freedom as the previous normal state we considered. In particular, we can consider a strong coupling T-IVC normal state with projector of the form:

$$P_{\nu=2} = \frac{1}{2}\left(1 + \eta_x\rho_x\right) \tag{S31}$$

In contrast to the case for a normal state with coexisting T-IVC and spin-polarized order, there are now more possible pairing options and singlet pairing is once again possible. We can classify the possibilities as pairings which are triplet, singlet, and by IRs of the point group. We find the possible pairings include triplet A_1 and A_2 pairings and singlet B_1 and B_2 pairings with:

$$\Delta_{\boldsymbol{k}} \sim P_{\nu=2}s_x\eta_z\rho_z \quad \Delta_{\boldsymbol{k}} \sim P_{\nu=2}s_0\eta_z\rho_z \tag{S32}$$

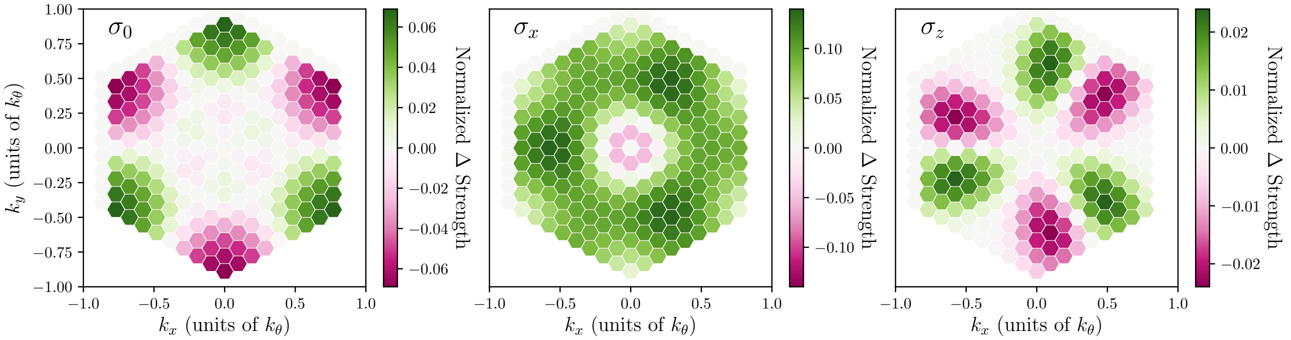

FIG. S1: Highest eigenvalue pairing obtained from linearized gap equation at $T = 16$ K, for SLP- and T-IVC fluctuations. The pairing transforms under the B_2 representation of the point group.

triplet B_1 and B_2 pairings and singlet versions of our A_1 and A_2 states with:

$$\Delta_{\boldsymbol{k}} \sim P_{\nu=2} s_x \eta_0 \rho_0 \quad \Delta_{\boldsymbol{k}} \sim P_{\nu=2} s_0 \eta_0 \rho_0 \tag{S33}$$

and triplet E_1 pairing and singlet E_2 pairing with:

$$\Delta_{\boldsymbol{k}} \sim P_{\nu=2} s_x (\eta_0 \rho_x, \eta_z \rho_y) \quad \Delta_{\boldsymbol{k}} \sim P_{\nu=2} s_0 (\eta_0 \rho_x, \eta_z \rho_y) \tag{S34}$$

Of the above, the only options which are not enforced to have a sign change are our purely inter-band A_2 triplet pairing, the A_1 singlet pairing, and the E_2 singlet pairing. Since these pairings do not have a sign change, they are the only possible candidates for the criterion around Eq. (7) of the main text and we have enumerated the possible pairing glues for these s-wave states in Table S3.

$\Delta_{\boldsymbol{k}}$	IR of D_6	T-IVC/A_1 phonon $\eta_x \rho_x$	quantum spin Hall $\boldsymbol{s} \eta_z \rho_z$	Spin polarized $\boldsymbol{s}$	N-IVC $\eta_x(\rho_0, \eta_z \rho_z)$	quantum Hall $\eta_z \rho_z$
$P_{\nu=2}\,(s_x \eta_z \rho_z \delta_{\boldsymbol{k}})$	A_2 (triplet)	✓	✗	✓	✗	✗
$P_{\nu=2}\,(s_0 \eta_0 \rho_0 \delta_{\boldsymbol{k}})$	A_1 (singlet)	✓	✓	✗	✓	✗
$P_{\nu=2}\,(s_0(\eta_0 \rho_x, \eta_z \rho_y))$	E_2 (singlet)	✓	✗	✗	✓	✓

TABLE S3: We list the possible pairing glues which are compatible with a T-IVC normal state (ie the interactions survive projection to the space of the upper spin-degenerate T-IVC bands). We denote interactions which will generate an attractive interaction with a ✓and interactions which will generate a repulsive interaction with a ✗.

We find in this case that all of the pairing glues which are attractive for our A_2 triplet pairing are also attractive for one of the singlet pairings, except for spin fluctuations. Therefore, we can say that if the pairing is triplet for a spin-degenerate T-IVC normal state, the leading instability is likely to be our A_2 pairing provided the pairing glue interaction is sufficiently strong and spin fluctuations may play an important role in energetically favoring this state. In this case, we expect that the phenomenology of Bogoliubov Fermi surfaces and a nodal to gapped transition as a function of interaction strength will again apply.

SI F. MORE SUPERCONDUCTING INSTABILITIES

In this appendix, we will discuss the superconducting instabilities we find beyond the A_2 and B_1 states shown in Figs. 2 and 3 of the main text and focus on the other leading instabilities we find in the presence of fluctuations of different particle hole orders. For SLP$-$ fluctuations, we find the B_2 state can be favored over the B_1 when the strength of T-IVC fluctuations are on the same order as SLP$-$ fluctuations, as shown in Fig. 4. We show the B_2 state for parameter value $\theta_{fluc.} \simeq \frac{\pi}{4}$ in Fig. S1. For N-IVC fluctuations as well as for SLP$-$ fluctuations, we find the E_2 is the leading instability, as shown in Fig. 6. We show the two components of the E_2 state for parameter value $\theta_{fluc.} \simeq \frac{\pi}{2}$ in Fig. 4.

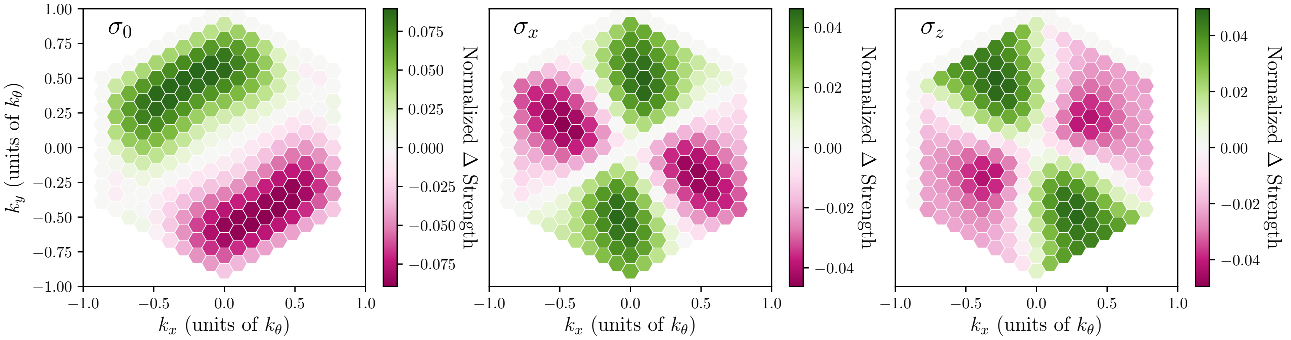

FIG. S2: First component of the highest eigenvalue pairing obtained from linearized gap equation at $T = 16$ K for just N-IVC fluctuations. The pairing transforms under the E_1 representation of the point group. The component shown here is degenerate with the other basis functions which transform under C_3 symmetry shown in Fig. S3.

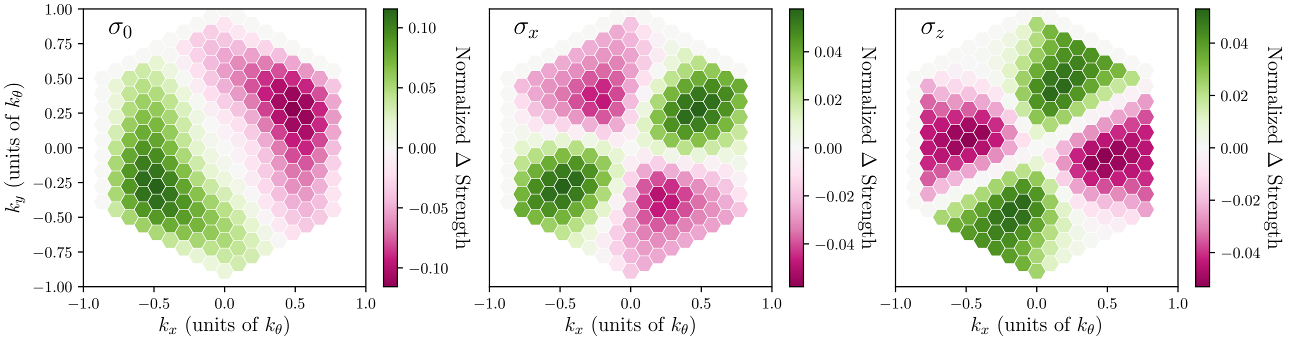

FIG. S3: Second component of the highest eigenvalue pairing obtained from linearized gap equation at $T = 16$ K for just N-IVC fluctuations. The pairing transforms under the E_1 representation of the point group. The component shown here is degenerate with the other basis functions which transform under C_3 symmetry shown in Fig. S2.

We point out that each component of the E_1 pairing shown in Figs. S2 and S3 may by themselves be nodal, assuming the pieces of each pairing which are proportional to σ_x in band space are smaller than the band splitting. In general, we expect the lowest energy pairing at $T = 0$ will be the chiral E_1 state which would be fully gapped; however, in the presence of sufficient strain, a single basis function of the E_1 pairing can be favored over the chiral state, offering another route to nodal superconductivity in the presence of N-IVC fluctuations.

---

[1] M. S. Scheurer and R. Samajdar, "Pairing in graphene-based moiré superlattices," Phys. Rev. Research **2**, 033062 (2020).
[2] M. Christos, S. Sachdev, and M. S. Scheurer, "Correlated Insulators, Semimetals, and Superconductivity in Twisted Trilayer Graphene," Phys. Rev. X **12**, 021018 (2022), arXiv:2106.02063 [cond-mat.str-el].
[3] E. Morissette, J.-X. Lin, D. Sun, L. Zhang, S. Liu, D. Rhodes, K. Watanabe, T. Taniguchi, J. Hone, J. Pollanen, M. S. Scheurer, M. Lilly, A. Mounce, and J. I. A. Li, "Dirac revivals drive a resonance response in twisted bilayer graphene," Nature Physics (2023), 10.1038/s41567-023-02060-0.
[4] R. Bistritzer and A. H. MacDonald, "Moiré bands in twisted double-layer graphene," Proceedings of the National Academy of Sciences **108**, 12233 (2011).
[5] E. Khalaf, A. J. Kruchkov, G. Tarnopolsky, and A. Vishwanath, "Magic angle hierarchy in twisted graphene multilayers," Phys. Rev. B **100** (2019), 10.1103/physrevb.100.085109.
[6] U. Zondiner, A. Rozen, D. Rodan-Legrain, Y. Cao, R. Queiroz, T. Taniguchi, K. Watanabe, Y. Oreg, F. von Oppen, A. Stern, E. Berg, P. Jarillo-Herrero, and S. Ilani, "Cascade of phase transitions and dirac revivals in magic-angle graphene," Nature **582**, 203 (2020).
[7] D. Wong, K. P. Nuckolls, M. Oh, B. Lian, Y. Xie, S. Jeon, K. Watanabe, T. Taniguchi, B. A. Bernevig, and A. Yazdani, "Cascade of electronic transitions in magic-angle twisted bilayer graphene," Nature **582**, 198 (2020).
[8] R. Samajdar, M. S. Scheurer, S. Turkel, C. Rubio-Verdú, A. N. Pasupathy, J. W. F. Venderbos, and R. M. Fernandes, "Electric-field-tunable electronic nematic order in twisted double-bilayer graphene," 2D Materials **8**, 034005 (2021).

[9] K. P. Nuckolls, R. L. Lee, M. Oh, D. Wong, T. Soejima, J. P. Hong, D. Călugăru, J. Herzog-Arbeitman, B. A. Bernevig, K. Watanabe, T. Taniguchi, N. Regnault, M. P. Zaletel, and A. Yazdani, "Quantum textures of the many-body wavefunctions in magic-angle graphene," (2023).

[10] E. Lake, A. S. Patri, and T. Senthil, "Pairing symmetry of twisted bilayer graphene: A phenomenological synthesis," Phys. Rev. B **106** (2022), 10.1103/physrevb.106.104506.
